# Differentially expressed ncRNAs as key regulators in infection of human bronchial epithelial cells by the SARS-CoV-2 Delta variant

**DOI:** 10.1016/j.omtn.2025.102559

**Published:** 2025-05-14

**Authors:** Glory Ranches, Hubert Hackl, Viktoria Zaderer, Melanie Ploner, Wilfried Posch, Doris Wilflingseder, Kai Kummer, Alexander Hüttenhofer

**Affiliations:** 1 Institute of Pathology, Neuropathology and Molecular Pathology, Medical University of Innsbruck, Innsbruck, Austria; 2 Institute of Bioinformatics, Biocenter, Medical University of Innsbruck, Innsbruck, Austria; 3 Institute of Hygiene and Medical Microbiology, Medical University of Innsbruck, Innsbruck, Austria; 4 Division of Genomics and RNomics, Biocenter, Medical University Innsbruck, Innsbruck, Austria; 5 Ignaz Semmelweis Institute, Interuniversity Institute for Infection Research, University of Veterinary Medicine, Vienna, Austria; 6 Institute of Physiology, Medical University of Innsbruck, Innsbruck, Austria

**Keywords:** MT: Non-coding RNAs, SARS-CoV-2, non-coding RNAs, transcriptomic profile, virus variants, pathways

## Abstract

SARS-CoV-2 infection initiates complex interactions at mucosal barriers. In primary human bronchial epithelial cells, we investigated changes in the small RNA transcriptome induced by Delta variant infection. Thereby, we uncovered differential expression of a specific set of microRNAs (miRNAs), PIWI-interacting RNAs (piRNAs), small nucleolar RNAs (snoRNAs), vault RNAs, Y RNAs, and long intergenic non-coding RNAs (lincRNAs), which inhibit apoptosis while promoting cell proliferation and viral infection. Conversely, differential expression of 7SL, U2, and RPPH1 RNAs, as well as miR-155-5p and miR-27a-5p, was found to be involved in antiviral signaling. In addition, expression of the protein-coding genes *CXCL10*, *IFIT1*, *NCOA7*, *IFIT2*, *SIX3*, and *RPSA* was increased during infection. Interestingly, the ribosomal protein RPSA has recently been reported to also serve as a viral surface receptor promoting pro-inflammatory cytokine signaling. By investigating these differentially expressed genes also after Omicron BA.2 variant infection, we observed a significantly lower expression of the protein-coding genes *CXCL10*, *IFIT2*, and *ZC3HAV1*. In contrast, expression changes for the majority of non-coding RNAs (ncRNAs) were similar between Delta and Omicron variants with the exception of miR-155-5p and 5′-tRF^Glu(TTC)^, emphasizing their potential as biomarkers for disease severity. Our findings thus highlight distinct molecular responses in SARS-CoV-2-infected cells, revealing specific genes and ncRNAs involved in viral replication, immune response, and apoptosis.

## Graphical abstract

**Figure:**
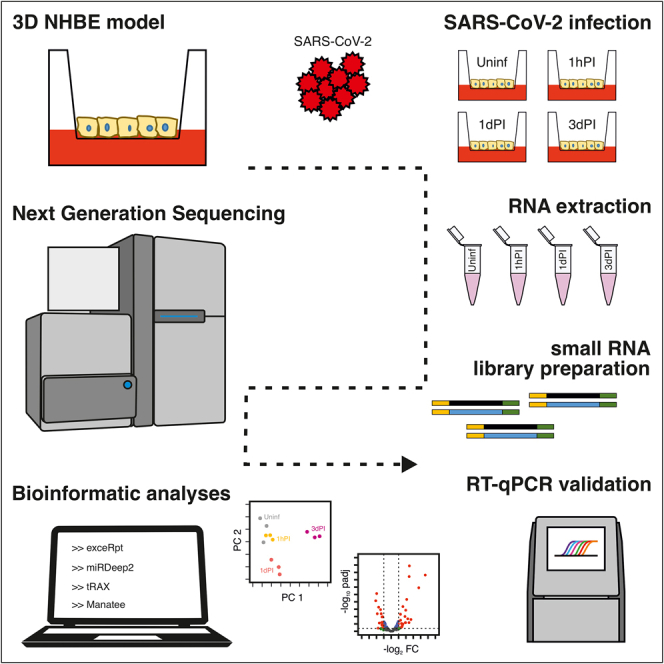

## Teaser

Wilflingseder, Kummer, Hüttenhofer, and colleagues highlighted alterations in non-coding RNA expression during SARS-CoV-2 infection. They demonstrate that distinct ncRNA groups regulate shared and unique molecular pathways, including apoptosis, immune response, and viral replication, revealing variant-specific molecular dynamics with potential utility as biomarkers for disease severity.

## Introduction

Coronaviruses (CoVs) are enveloped, positive-sense, single-stranded RNA viruses with a helical nucleocapsid and a diameter of approximately 120 nm.^1^ CoVs infect the respiratory tract and vary significantly in infection risk—e.g., some exert a mild course (e.g., HCoV-NL-63) causing the common cold, while others can be lethal (e.g., SARS-CoV-1, SARS-CoV-2, and MERS-CoV). Due to the contagiousness of SARS-CoV-2 and its rapid spread in March 2020, the World Health Organization declared COVID-19 as a global pandemic.

The continuous emergence of novel SARS-CoV-2 variants and their respective subvariants (e.g., Omicron BA.2/.4/.5; XBB.1.5.5; BQ.1.1; JN.1—a subvariant of BA.2.86; and KP.2, KP.3, and LB.1—subvariants of JN.1) has raised concerns about the efficacy of current SARS-CoV-2 vaccines despite their adaptation to Omicron subvariants BA.4, BA.5, and XBB.1.5.5.^2^^,^^3^ In particular, the novel Omicron JN.1 subvariants, which are very efficient at immune evasion, are highly contagious and potentially less susceptible to current vaccines.^4^^,^^5^ Thus, there is still an urgent need to understand viral entry and host cell mechanisms employed by the different SARS-CoV-2 variants in more detail.

To evaluate the very first encounter of SARS-CoV-2 variants with human epithelial tissues, 3D models of the human respiratory tract have been shown to be highly suitable cell systems.^6^^,^^7^^,^^8^ Understanding these early events of host-pathogen interactions in realistic human *in vitro* systems allows accelerated characterization of ongoing infection events at barrier sites. Thus, for our study, we used a highly differentiated primary, pseudostratified, mucus-producing, and ciliated human 3D tissue model infected with the SARS-CoV-2 Delta variant. Interestingly, recent reports suggest that SARS-CoV-2 infection is also associated with the disruption of epigenetic regulation. In particular, the groups of Zazhytska et al. and Kee et al. have shown that epigenetic regulation by the virus occurs via histone mimicry,^9^^,^^10^ a mechanism also previously described for other highly virulent viruses.^11^^,^^12^

In this study, we focused on early gene regulatory events within a well-defined, standardized human ciliated cell model during SARS-CoV-2 infection. In particular, we aimed to identify small non-coding RNAs (referred to as ncRNAs) involved in viral infection, since this class of RNA species has previously been shown to act as genetic switches, e.g., by regulating the expression of target mRNAs. Thereby, ncRNAs, such as microRNAs (miRNAs), target the 3′ untranslated region of mRNAs and thus downregulate their expression.^13^ In addition to miRNAs, numerous other ncRNAs have been shown in the past to be involved in the regulation of various aspects of gene expression such as transcription or translation, in particular in the context of viral infections.^14^

For the investigation of ncRNAs involved in various aspects of gene regulation, several studies have focused either on the analysis of the small ncRNA transcriptome, e.g., miRNAs, sized 21–23 nucleotides (nt), or on longer ncRNAs (lncRNAs), such as long intergenic non-coding RNAs (lincRNAs), which—by definition—exhibit sizes larger than 200 nt. Since we consider this to be a rather arbitrary classification, especially in the absence of any functional correlation, we aimed to investigate SARS-CoV-2 Delta-induced changes in the small ncRNA transcriptome of approximately 20–200 nt. In a previous study, we also investigated the small ncRNA transcriptome of Epstein-Barr virus (EBV)-infected human cells using this approach and were able to identify several cellular as well as virus-encoded ncRNAs, such as vault RNAs (vtRNAs), small nucleolar RNAs (snoRNAs) or miRNAs, which are used by EBV to promote infection of human target cells and thus propagation of EBV viral particles.^15^

Hence, we aimed to identify changes within the small ncRNA transcriptome involved in the infection of respiratory epithelial cells by the Delta variant for 1 h, 1 day, or 3 days, respectively, and compared the small ncRNA transcriptome of lung epithelial cells, as assessed by RNA sequencing (RNA-seq), with that of uninfected cells. For those differentially expressed small RNAs identified in Delta-infected cells, we also investigated by RT-qPCR and norther blot analysis whether these were similarly dysregulated in a second variant of SARS-CoV-2, the Omicron BA.2 strain.

## Results

### Increased infection of differentiated NHBE cultures by the Delta variant

**Figure 1:**
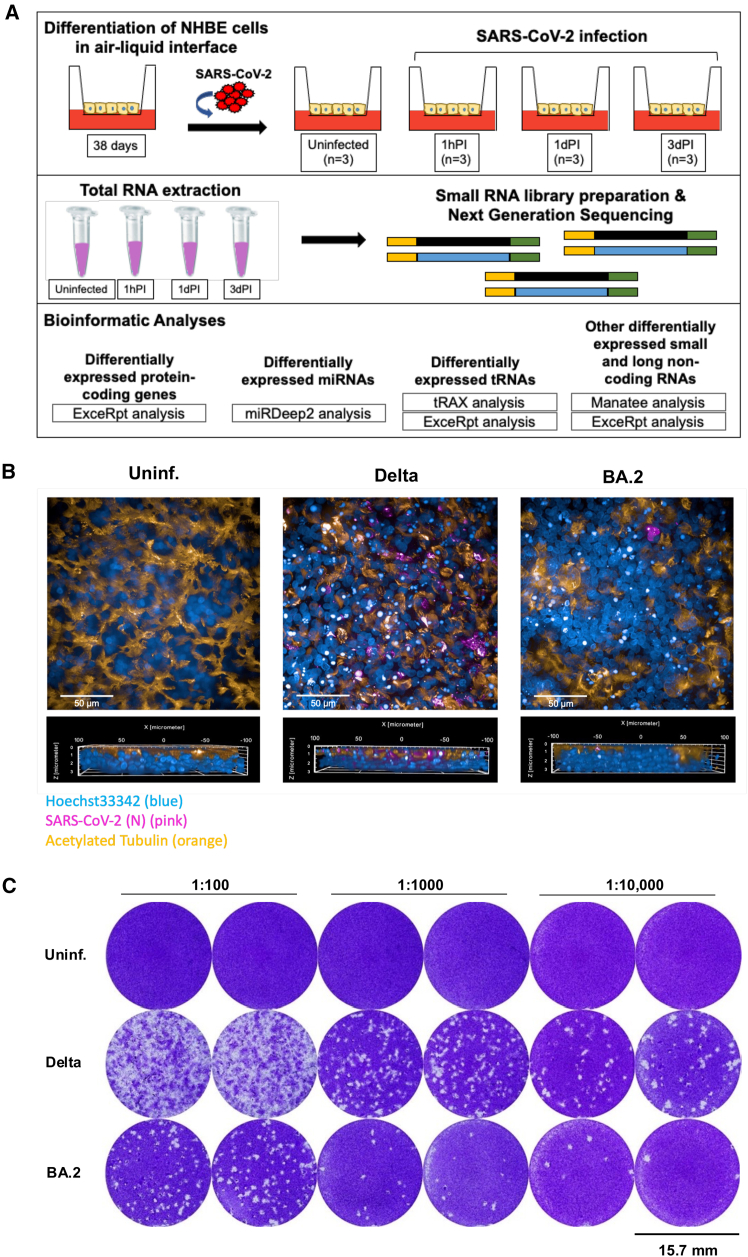
SARS-CoV-2 infection and characterization (A) Schematic diagram of SARS-CoV-2 (Delta) treatment of differentiated NHBE cells in air-liquid interface (ALI) culture system. Following post infection of cells for 1 h (1hPI), 24 h or 1 day post-infection (1dPI), and 72 h or 3 days post-infection (3dPI), cells were harvested and analyzed for total RNA extraction. Uninfected (Uninf.) cells were used as controls. Total RNA extracts were analyzed for small RNA-seq library preparation and RNA sequencing (RNA-seq). RNA-seq data were processed by employing specific data analysis pipelines to determine differentially expressed genes in the presence of SARS-CoV-2. (B) Representative images of NHBE cells, which were either Uninf. or infected with SARS-CoV-2 Delta or Omicron BA.2 for 72 h. Cells were fixed and stained for Hoechst (blue), SARS-CoV-2 (N) (pink), and acetylated tubulin (orange), and subsequently analyzed by confocal microscopy. Scale bars, 50 μm. (C) Productive infection of NHBE cells after 72 h following Delta (middle) or Omicron BA.2 (BA.2) (bottom) infection is illustrated. Uninf. NHBE cells (top) served as negative control. VeroE6/TMPRSS2/ACE2 were inoculated with supernatants of Uninf., Delta-, or BA.2-infected NHBE cultures at different dilutions (i.e., 1:100, 1:1,000 and 1:10,000). After 72 h, cells were stained using crystal violet and viral plaques were counted to analyze productive infection. Assays were performed at least 3 times and a representative experiment is depicted. Scale bars, 15.7 mm.

Normal human bronchial epithelial (NHBE) cells were cultured for 38 days under air-liquid interphase (ALI) prior to infection with SARS-CoV-2 Delta and Omicron BA.2 variants for up to 72 h (i.e., 3 days) (Figure 1A). As previously described, at this time point, a fully differentiated epithelial layer with basal cells, ciliated cells, mucus-producing cells, and high mucociliary clearance capacity is formed.^6^^,^^16^ To characterize and verify SARS-CoV-2 infection in NHBE cells, we subsequently performed immunocytochemical analysis. At 3 days post infection (3dPI), uninfected/mock-treated tissue cultures (Uninf.) displayed an intact pseudostratified epithelium.

Infection with the SARS-CoV-2 Delta strain (i.e., strain MOI 0.1 from which RNA-seq analysis was performed) resulted in multiple infection foci throughout the whole epithelium (Figure 1B, pink), and destruction of the tissue integrity as analyzed by staining of the ciliated border using acetylated tubulin (Figure 1B, orange) and fragmentation of nuclei (Hoechst) (Figure 1B, Uninf. vs. Delta). In contrast to the Delta strain, only superficial destruction and fragmentation of nuclei was observed in Omicron BA.2-infected cells (see the following text). In addition, ciliated borders were not as severely disrupted by the Omicron BA.2 virus compared to the Delta variant and showed a more similar phenotype to uninfected cultures (Figure 1B, BA.2 vs. Delta vs. Uninf.).

We also observed differences between Delta and Omicron BA.2 infection as shown by viral plaque assay (Figure 1C). After 72 h, VeroE6/TMPRSS2/ACE2 monolayers were inoculated with supernatants from Uninf., Delta, and Omicron BA.2-infected NHBE cells in three dilutions (i.e., 1:100, 1:1,000, and 1:10,000). Similar to the confocal microscopic analysis, the viral plaque assay also showed a higher infection in Delta-infected compared to Omicron BA.2-infected NHBE cells (Figure 1C).

### RNA-seq analysis of the small RNA transcriptome from uninfected NHBE cells or NHBE cells infected with the Delta variant

We next isolated total RNA from uninfected or SARS-CoV-2 Delta-infected NHBE cells at 1 h post infection (1hPI), 24 h post infection (1dPI) or 72 h post infection (3dPI) (Figure 1A). Prior to RNA-seq analysis, we assessed the quality and integrity of total RNA extracts from NHBE cells by Bioanalyzer analysis and included all samples with RNA integrity number values ≥8 (Figure S1). We then generated cDNA libraries encoding RNAs sized 20–200 nt using the Lexogen small RNA kit and analyzed the sequences using Illumina sequencing technology (GENEWIZ, Germany).

Based on the chosen RNA-seq approach, we observed ncRNA species less than 200 nt in length from trimmed and pre-processed sequence reads, consistent with our cDNA library generation strategy. The distribution of sequence reads was found at ∼20–30 nt read length, but the majority of peaks or fragments were determined from ∼60 nt to ∼95 nt read length, whereas the highest percentage of reads was displayed at ∼150 nt read length (Figure S2).

Several algorithms are available for the analysis of RNA-seq data to identify expression of ncRNA genes, and each of these algorithms has advantages and disadvantages for sequence analysis.^17^ Therefore, we employed several different approaches to identify significantly differentially expressed ncRNAs in Delta-infected NHBE cells (Figure 1A), providing more precise and accurate results. Alignment was performed using the extracellular RNA processing tool (exceRpt) pipeline for protein-coding genes,^18^ miRdeep2 for miRNAs,^19^ exceRpt and tRNA analysis of expression (tRAX) for tRNAs,^20^ and a combination of Manatee^21^ and the exceRpt pipeline for other small and lncRNAs.

### Dysregulated expression of protein-coding genes in SARS-CoV-2 Delta-infected cells

Initially, we employed the exceRpt analysis, which involves multiple cascades of computational filters and alignments and provides an appropriate level of confidence in a given set of annotated RNAs. This toolkit has been used for small RNA-seq data analysis, as it can generate quality control reports and abundance estimates for RNA biotypes.^18^ In addition, this analysis also identified differentially expressed protein-coding genes that, due to our RNA-seq approach described previously, are likely to resemble shorter expressed sequence tags (ESTs) of corresponding full-length mRNAs, as observed for ncRNAs.

**Figure 2:**
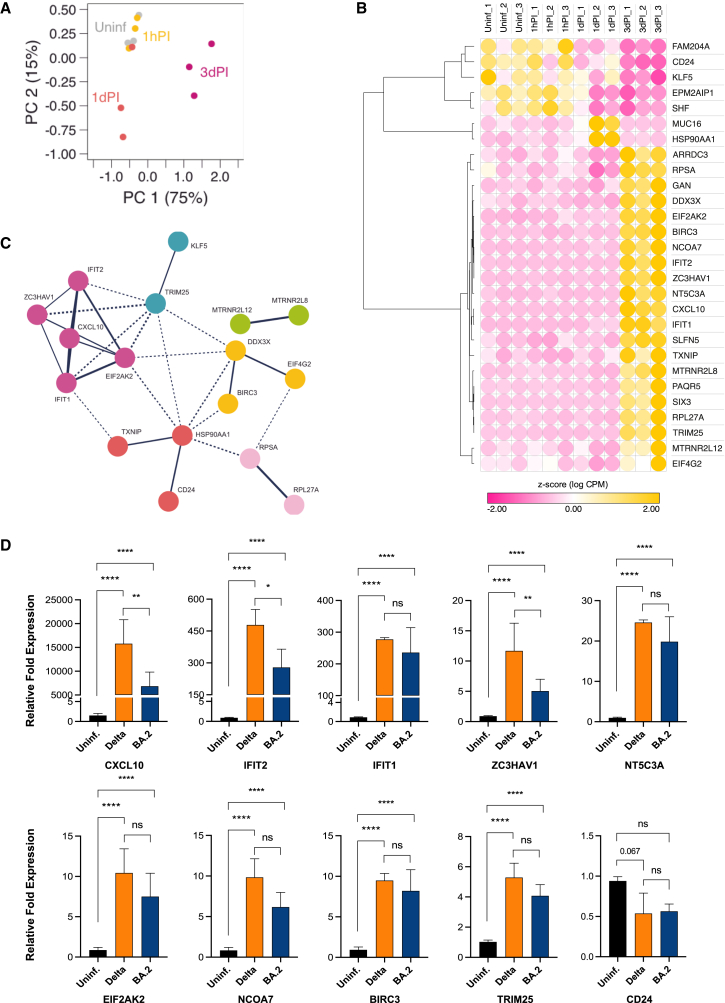
Differentially expressed protein-coding genes induced by SARS-CoV-2 in NHBE cells (A) Principal-component analysis of differentially expressed protein-coding genes in NHBE cells upon SARS-CoV-2 (Delta) infection following 1 h post-infection (1hPI), 24 h or 1 day post-infection (1dPI), and 72 h or 3 days post-infection (3dPI). Uninfected (Uninf.) NHBE cells were used as control group. (B) Heatmap of differentially expressed protein-coding genes shown as relative *Z* score, which indicates the normalized expression levels of each group relative to the mean. Positive values (yellow) indicate high expression and negative values (pink) represent low expression per gene. (C) Protein-protein interaction network for differentially expressed protein-coding genes. Clustering was performed using MCL clustering with inflation parameter set to 3. Disconnected nodes are hidden from the network. (D) RT-qPCR validation of selected protein-coding gene candidates in (B) using Uninf. and SARS-CoV-2-(Delta or Omicron BA.2)-infected NHBE cells at 3dPI. Bar graphs indicate mean ± standard deviation of fold change in expression of gene candidates in SARS-CoV-2-infected cells relative to Uninf. cells. Two-way ANOVA followed by Tukey’s post-hoc test was performed using normalized Ct values. The analysis was performed in triplicates using at least three independent samples. ∗*p* < 0.05, ∗∗*p* < 0.01, and ∗∗∗∗*p* < 0.0001; and ns, not significant.

Upon applying the exceRpt algorithm to our sequence data, a clear separation of gene expression patterns between 3dPI and Uninf., 1hPI, and 1dPI time points was observed by principal-component analysis (PCA) of the normalized RNA-seq data (Figure 2A). While genes expressed at Uninf. and 1hPI time points showed similar expression patterns as shown by clustering of these samples in the PCA, samples from 1dPI already showed altered gene expression patterns. In addition, gene expression from 3dPI samples was clearly separated from all other time points (Figure 2A).

We identified 28 significantly dysregulated protein-coding genes (Figure 2B; Table S1). Notably, in our analyses, we observed increased expression of previously described viral infection-related genes,^22^ including *CXCL10*, *EIF2AK2*, *BIRC3*, as well as antiviral genes and genes involved in pathogen response, such as *IFIT1*, *IFIT2*, *ZC3HAV1*, *DDX3X* (*DDX3*), *SLFN5*, *TRIM25*, *NCOA7*, *SIX3*, *NT5C3A*, and *PAQR5* (Figure 2B). Downregulated gene candidates included *FAM204A*, *CD24*, *KLF5*, *EPM2AIP1*, *SHF*, *MUC16*, and *HSP90AA1* (Figure 2B) especially at 3dPI (with the exception of *MUC16* and *HSP90AA1*, which were upregulated at 1dPI). Figure 2C shows a protein-protein network representation of marker gene clusters during Delta infection at 3dPI, reflecting a “cytokine storm” phenomenon as indicated by an elevated expression of *CXCL10* and associated interferon (IFN)-stimulated genes (*IFIT1*, *IFIT2*, *ZC3HAV1*, and *EIF2AK2*; purple cluster), transcription regulation (*DDX3X*, *EIF4G2*, and *BIRC3*; yellow cluster), innate immune defense against viruses (*TRIM25* and *KLF5*; blue cluster), anti-apoptotic functions (*MTRNR2L12* and *MTRNR2L8*; green cluster), downmodulation of exosome pathway (CD24 and HSP90AA; orange cluster), or virus attachment (*RPSA* and *RPL27A*; rose cluster) (Tables S2 and S3).

Using RT-qPCR, we additionally validated a number of protein-coding gene candidates that were consistently differentially up- or downregulated in the presence of the Delta variant in a pseudostratified epithelial model (Figure 2D). The upregulated genes (highest to lowest) relative to control or uninfected samples include *CXCL10*, *IFIT2*, *IFIT1*, *NT5C3A*, *ZC3HAV1*, *EIF2AK2*, *NCOA7*, *BIRC3*, and *TRIM25*, respectively (Figure 2D), all of which are associated with inflammatory and antiviral immune responses.

### Analysis of Delta-induced differentially expressed RNAs in the Omicron BA.2 variant

In the following, we evaluated whether the differential expression of these genes and identified small RNAs (see the following text) during infection with the Delta variant could also be found in the Omicron BA.2 variant. Consistently, by this analysis we observed a significant dysregulation of the expression of all ten gene candidates in Omicron BA.2-infected cells relative to uninfected cells (Figure 2D), although the downregulation of *CD24* did not reach statistical significance. Among the validated gene candidates, we identified three protein-coding genes, i.e., *CXCL10*, *IFIT2*, and *ZC3HAV1*, whose expression was significantly lower in Omicron BA.2-infected cells compared to the Delta strain (Figure 2D).

Using expression profiling of protein-coding genes by RNA-seq and the validation of differentially regulated genes by RT-qPCR in primary NHBE cells, we identified corresponding expression patterns of the newly identified SARS-CoV-2 interaction partners as compared to previous transcriptomic and proteomic analyses from COVID-19 patients and other *in vitro* studies.^23^^,^^24^^,^^25^ Hence, these results fully demonstrate the suitability of our highly differentiated and standardized *in vitro* model system. In the following, we therefore investigated changes in the small ncRNA transcriptome response upon viral infection to unveil a more detailed mechanistic understanding of the initial steps in COVID-19 pathogenesis at the level of regulatory ncRNAs.

### Differentially expressed miRNAs and target pathway analysis

One of the most thoroughly studied classes of ncRNAs is miRNAs, which are 18–25 nt-long single-stranded ncRNA molecules. miRNAs are potent modulators of gene expression, capable of targeting a substantial number of genes for translational repression or target mRNA degradation. In this study, we employed the miRDeep2 algorithm as a specific computational analysis tool for miRNAs.^19^ Clustering analysis of the normalized RNA-seq data revealed a clear separation of the miRNA expression pattern between the Uninf., 1dPI, and 3dPI groups (Figure S3), while samples from the 1hPI group clustered closely with the Uninf. group, suggesting a longer latency for virally induced miRNA gene expression changes.

**Figure 3:**
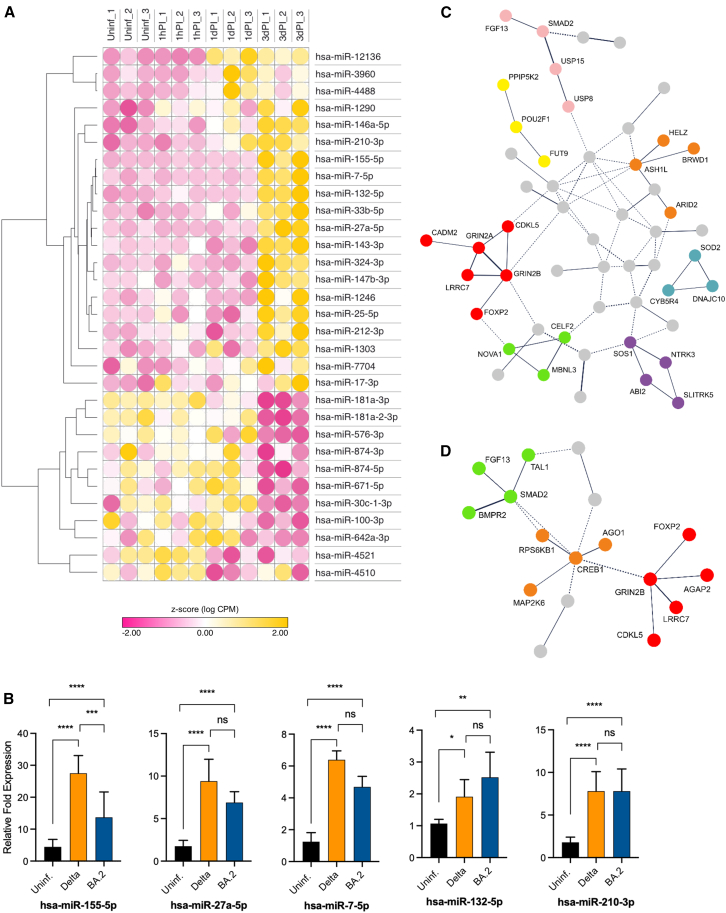
Significantly dysregulated miRNAs in NHBE cells upon SARS-CoV-2 infection (A) Heatmap of dysregulated miRNA candidates in SARS-CoV-2 (Delta)-infected NHBE cells following 1 h post-infection (1hPI), 24 h or 1 day post-infection (1dPI), and 72 h or 3 days post-infection (3dPI). The relative *Z* score values, which indicate the normalized expression of each group relative to the mean, are shown. Positive values (yellow) indicate high expression and negative values (pink) represents low expression. (B) RT-qPCR validation of selected differentially expressed miRNA candidates in uninfected (Uninf.) and SARS-CoV-2-(Delta or Omicron BA.2)-infected NHBE cells at 3dPI. The experiment was performed using at least three independent samples (*n* = 3) for each group and the analysis was carried out in triplicates. Bar graphs indicate mean ± standard deviation of fold change in expression of selected miRNA candidates in SARS-CoV-2-infected cells relative to Uninf. cells. Two-way ANOVA followed by Tukey’s post-hoc test was performed using normalized Ct values. The experiment was performed using at least three independent samples. ∗*p* < 0.05, ∗∗*p* < 0.01, ∗∗∗*p* < 0.001, and ∗∗∗∗*p* < 0.0001; and ns, not significant. (C and D) Protein-protein interaction (PPI) networks of main genes predicted to be targeted by at least 50% of significantly upregulated (C) or downregulated (D) miRNAs. Clustering performed using MCL clustering with inflation parameter set to 3. Disconnected nodes are hidden from the network.

Differential expression analysis revealed a total of 31 dysregulated miRNA candidates, of which 65% were upregulated (Figure 3A), with the majority of miRNAs showing increased expression at 3dPI (e.g., hsa-miR-155-5p, hsa-miR-27a-5p, and hsa-miR-132-5p), and only for a few the expression was already increased at 1dPI (e.g., hsa-miR-3960, hsa-miR-4488, and hsa-miR-12136). The downregulated expression of miRNAs also showed the strongest regulation at 3dPI (e.g., hsa-miR-874-5p, hsa-miR-100-3p, and hsa-miR-181a-1-3p; Table S4).

We selected five differentially expressed miRNAs for RT-qPCR validation, all of which showed an increased abundance during both Delta and Omicron BA.2 infection (Figure 3B). In contrast, the expression of hsa-miR-155-5p was observed to be significantly higher in Delta-infected cells compared to Omicron BA.2-infected cells.

According to the canonical miRNA pathway, upregulation of miRNAs results in the downregulation of mRNA target genes, whereas downregulation of miRNAs leads to the upregulation of gene targets. Therefore, we performed a target prediction analysis for counterregulated pairs of differentially expressed miRNAs and mRNAs. Interestingly, 75% of the differentially expressed mRNAs (3 out of 4 downregulated mRNAs and 16 out of 21 upregulated mRNAs) were predicted to be targeted by at least 50% of the counterregulated miRNAs (data not shown).

Since multiple coregulated miRNAs may share targets associated with common regulatory pathways, we performed unbiased target prediction analyses separately for up- and downregulate miRNAs. The upregulated miRNAs shared 118 common mRNA targets that were predicted to be targeted by at least 50% of the dysregulated miRNAs. Notably, *NFAT5* (nuclear factor of activated T cells 5), *SMAD2* (SMAD family member 2), *GRIN2B* (glutamate ionotropic receptor NMDA type subunit 2B), and *ZBTB20* (zinc finger and BTB domain containing 20) were among the genes targeted by these miRNAs. *NFAT5*, *SMAD2*, and *ZBTB20* are transcription factors known for their roles in regulating gene expression in response to cellular stress, transforming growth factor β (TGF-β) signaling and apoptosis regulation, and immune responses, respectively.^26^^,^^27^^,^^28^ Consistent with this, protein-protein interaction (PPI) and pathway analyses of the targeted genes revealed chromosomal rearrangement, ubiquitin-like protein (Ubl) conjugation, and alternative splicing pathways (entire PPI network; Figure 3C), Rho and Rac GTPase activity (violet cluster), regulation of alternative mRNA splicing (green cluster), and TGF-β signaling (rose cluster) as enriched pathways (Tables S5 and S6).

For the downregulated miRNAs, 45 mRNAs were predicted to be targeted by at least 50% of the miRNAs. Pathways shared by these gene targets included alternative splicing (entire PPI network; Figure 3D), chromosomal rearrangement (red cluster), MECP2 signaling, virus infection pathways, tumor necrosis factor and interleukin-related pathways (orange cluster), as well as TGF-β signaling (green cluster), according to PPI and pathway analysis (Tables S7 and S8). Interestingly, the majority of these pathways show associations with apoptotic mechanisms, as both intrinsic and extrinsic apoptotic pathways are tightly regulated by ubiquitination of associated proteins.^29^

### Differentially expressed piRNAs

PIWI-interacting RNAs (piRNAs) are single-stranded RNAs of 21–35 nt in size that have been reported to be involved in transcriptional silencing through silencing of transposable elements^30^ and mRNA elimination via a piRNA-induced silencing complex.^31^ In addition, some piRNAs have been shown to activate target mRNAs via imperfect base-pairing interactions.^32^ Recently, there has been increasing evidence linking piRNAs to cell proliferation and apoptosis, demonstrating a dual role for piRNAs with both pro- and anti-apoptotic effects.^33^

**Figure 4:**
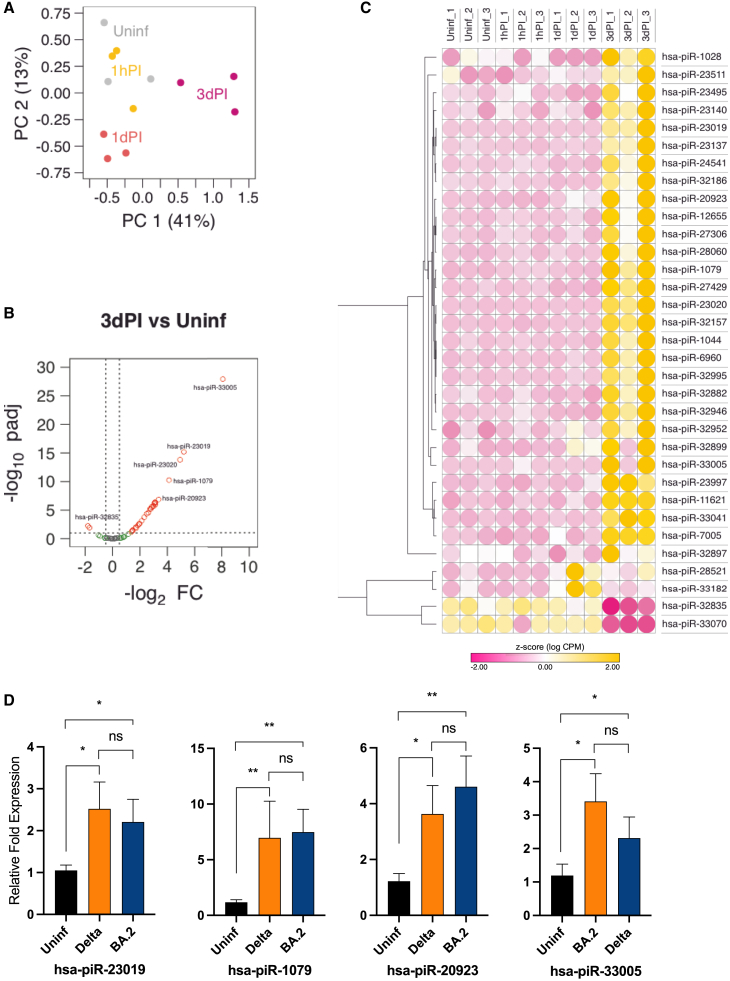
Differential expression of piRNAs observed in NHBE cells upon SARS-CoV-2 infection (A) Principal-component analysis of differentially expressed piRNAs in uninfected (Uninf.) and SARS-CoV-2 (Delta)-infected NHBE cells following 1 h post-infection (1hPI), 24 h or 1 day post-infection (1dPI), and 72 h or 3 days post-infection (3dPI). (B) Volcano plot of −log 10 *p* values adjusted (*p*adj) versus log_2_ fold change (FC) for piRNAs in infected NHBE cells (3dPI) compared to Uninf. cells. Significantly differentially expressed genes are highlighted in red dots. (C) Heatmap of dysregulated piRNA candidates (as in B), which is indicated by relative *Z* score values, showing the normalized expression levels of each group relative to the mean. Positive values (yellow) indicate high expression and negative values (pink) represent low expression. (D) RT-qPCR analysis of selected differentially expressed piRNA candidates in (C) using SARS-CoV-2 (Delta or Omicron BA.2)-infected NHBE cells (3dPI) compared with Uninf. group. The experiment was carried out using at least three independent samples (*n* = 3) for each group and the analysis was performed in triplicates for each sample. Bar graphs indicate mean ± standard deviation of fold change in expression of selected piRNA candidates in SARS-CoV-2-infected cells relative to Uninf. cells. Two-way ANOVA followed by Tukey’s post-hoc test was performed using normalized Ct values. ∗*p* < 0.05, ∗∗*p* < 0.01, ∗∗∗*p* < 0.001, and ∗∗∗∗*p* < 0.0001; and ns, not significant. The experiment was carried out using at least three independent samples.

In our analysis, we showed three distinct clusters of piRNA expression, separating Uninf./1hPI, 1dPI, and 3dPI groups by PCA (Figure 4A). Using Manatee data analysis, we identified a total of 33 differentially expressed piRNAs (Figures 4B and 4C) and found that the vast majority of piRNAs were upregulated, with hsa-piR-33005, hsa-piR-23019, and hsa-piR-23020 showing the highest increase in gene expression at 3dPI relative to the Uninf. group (Table S9). RT-qPCR validation of four selected piRNAs confirmed SARS-CoV-2 (i.e., Delta and Omicron BA.2) infection-induced upregulation (Figure 4D). Only two piRNAs, i.e., hsa-piR-32835 and hsa-piR-33070, showed significant downregulation in our sequencing approach (Table S9), although this differential expression could not be validated using RT-qPCR (data not shown).

One of the few curated databases for piRNAs is piRNAdb, which also includes predicted (mRNA or lincRNA) targets of piRNAs, is based on piRNA sequences overlapping with protein-coding and non-coding regions of transcripts.^34^ While no target information is available for the majority of dysregulated piRNAs, a number of interesting piRNA::mRNA associations emerged (Table S10), and their putative targets are related to the regulation of apoptosis and antiviral responses.

### Differentially expressed tRNAs and tRNA fragments

**Figure 5:**
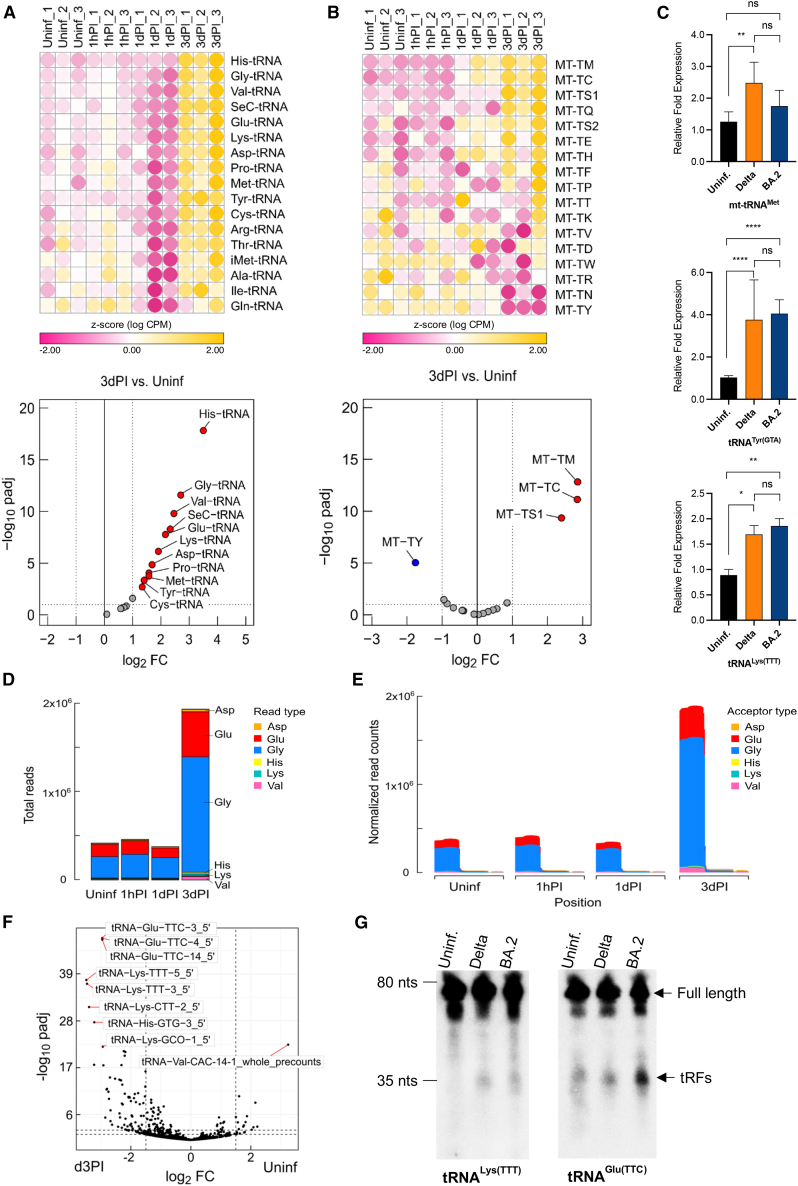
Identification of differentially expressed tRNA genes in NHBE cells during the onset of SARS-CoV-2 infection by exceRpt analysis and tRFs using tRAX analysis (A) Heatmap (left) and volcano plot (right) analyses of significantly dysregulated nuclear-encoded tRNAs in SARS-CoV-2 (Delta)-infected NHBE cells for 1 h post-infection (1hPI), 24 h or 1 day post-infection (1dPI), or 72 h or 3 days post-infection (3dPI). Uninfected (Uninf.) cells were used as controls. For the heatmap, the normalized expression levels of each group relative to the mean are indicated by *Z* score values. Positive values (yellow) indicate high expression, whereas negative values (pink) indicate low expression. Log2 fold change versus −log10 *p* value of tRNA candidates in 3dPI NHBE cells versus the control (Uninf.) group are shown for the volcano plot. (B) (Left ) Heatmap analysis of differentially expressed mt-tRNAs in SARS-CoV-2 (Delta)-infected NHBE cells (1hPI, 1dPI, and 3dPI) relative to Uninf. cells (as in A) based on *Z* score values. Uninf. cells were used as controls. Yellow indicates high expression; whereas, pink indicates low expression. (Right) Volcano plot analysis of log2 fold change versus −log10 *p* value of mt-tRNAs candidates in 3dPI NHBE cells versus the control or Uninf. group. (C)Validation of selected tRNA candidates (in A and B) in Uninf. and SARS-CoV-2 (Delta or Omicron BA.2)-infected NHBE cells (3dPI) by RT-qPCR. The experiment was performed using three independent samples (*n* = 3) for each group and the analysis was done in triplicates for each sample. Bar graphs indicate mean ± standard deviation of fold change in expression of selected tRNA candidates in SARS-CoV-2-infected cells relative to Uninf. cells. Two-way ANOVA followed by Tukey’s post-hoc test was performed using normalized Ct values. ∗*p* < 0.05, ∗∗*p* < 0.01, and ∗∗∗∗*p* < 0.0001; and ns, not significant. (D) Distribution of most abundant nuclear-encoded tRNA gene in NHBE cells, which were either uninfected or infected with SARS-CoV-2 Delta variant for 1 h post-infection (1hPI), 24 h or 1 day post-infection (1dPI), or 72 h or 3 days post-infection (3dPI), based on total reads. (E) Read coverage analysis of tRNA species in Uninf. and infected NHBE cells as in (D), showing the overall distribution of fragments at the 5′ or 3′ position based on normalized read counts. (F) Volcano plot of log2 fold change values versus adjusted *p* values of tRNAs, showing differentially expressed tRNA isotypes in fragment, full length or other form. (G) Northern blot analysis of selected tRF candidates. An equal amount of total RNA extracts (pooled from four independent samples) derived from Uninf. and infected NHBE cells (3dPI) with SARS-CoV-2 Delta or Omicron BA.2 (BA.2) were used in the analysis.

We first assessed the expression of tRNAs in SARS-CoV-2 Delta-infected NHBE cells using the exceRpt algorithm. As shown in the heatmap and volcano plot (Figures 5A and 5B, left and right, respectively), a total of 26 differentially expressed tRNA genes were identified, of which 11 nuclear-encoded tRNA genes and 4 mitochondrially encoded tRNAs were differentially expressed at 3dPI relative to the Uninf. group (Table S11; Figures 5A and 5B, respectively). Among the nuclear-encoded tRNA candidates, tRNA^His^, tRNA^Gly^, tRNA^Sec^, tRNA^Val^, tRNA^Glu^, and tRNA^Lys^ were the most upregulated species in SARS-CoV-2-infected cells (Figure 5A; Table S11). For mitochondrial tRNA gene candidates, mt-tRNA^Met^ (MT-TM), mt-tRNA^Cys^ (MT-TC), and mt-tRNA^Ser1^ (MT-TS1) are among the most abundant tRNA species upon viral infection, while mt-tRNA^Tyr^ (MT-TY) was the most downregulated species in Delta-infected NHBE cells compared to uninfected cells (Figure 5B; Table S11).

Using RT-qPCR, we validated the differential expression of selected tRNA candidates and showed that the abundance of tRNA^Tyr^, tRNA^Lys^, and MT-TM was consistently increased in Delta-infected NHBE cells compared to uninfected cells (Figure 5C). We also demonstrated that the abundance of these candidate genes, i.e., tRNA^Tyr^ and tRNA^Lys^, was increased to a similar extent in the Omicron BA.2-infected NHBE cells, except for the mitochondrial tRNA^Met^, which was not upregulated after Omicron BA.2 infection (Figure 5C). RT-qPCR validation of the MT-TY did show a slight trend toward downregulation, although not significant (data not shown).

In addition to full-length tRNAs, which are generally between 70 and 95 nt in size, shorter fragments of tRNAs (tRNA-derived fragments referred to as tRFs or tRNA halves) have recently been identified, which have been shown to represent a new class of regulatory ncRNAs with distinct biological functions in cancers and stress-induced diseases.^35^

Since tRFs have also been implicated in viral infection,^36^ we further analyzed the RNA-seq data employing the tRAX algorithm to specifically analyze the species of specific tRNA-derived fragments or full-length tRNAs. The tRAX software specifically aligns sequence reads to tRNA transcripts and allows mapping of reads to multiple transcripts and gene loci, thus providing accurate results on the aligned reads whether they are transcript-specific, isodecoder-specific, isotype-specific, or non-specific tRNAs.

Based on the abundance or total tRNA reads, we showed that tRNA^Gly^, tRNA^Glu^, tRNA^Val^, tRNA^Asp^, tRNA^Lys^, and tRNA^His^ are the most predominantly expressed tRNA species in Delta-infected NHBE cells after three days of infection (3dPI) as compared to the Uninf., 1hPI, or 1dPI group (Figure 5D). Importantly, we showed that the majority of these tRNA species were present as 5′-tRNA fragments (Figure 5E), consistent with previous reports^37^ based on the read length distribution and coverage of normalized read counts of tRNA species from each group. In particular, the abundance of 5′-tRFs was strikingly more pronounced in 3dPI compared to all other groups (i.e., Uninf., 1hPI, and 1dPI) (Figure 5E). By comparing Delta-infected (3dPI) versus Uninf. NHBE cultures, we identified several significantly upregulated 5′-fragments of tRNAs, predominantly encoding various gene copies of 5′-tRNA^Glu(TTC)^, 5′-tRNA^Lys(TTT/CTT)^, and 5′-tRNA^His(GTG)^, as well as downregulated 5′-tRNA^Val(CAC)^ (Figure 5F; Table S12).

To investigate whether the candidate tRNAs were indeed present as fragments or full-length tRNAs, we analyzed their expressions and sizes by northern blot analysis. Our analysis showed that most tRNA species were present as full-length tRNAs. For shorter sequence reads identified by the tRAX algorithm, we predominantly identified 5′-tRFs (see the following text), which have previously been shown to be involved in the regulation of gene expression (Figure 5E).^38^ However, the lack of validation of most of these 5′-tRFs by northern blot analysis, in contrast to next generation sequencing (NGS) analysis, may be due to their general low abundance. This discrepancy between the low abundance of tRFs as assessed by northern blot analysis versus NGS analysis might be resolved taken into consideration that in our previous studies, we have shown that NGS analysis considerably favored reverse transcription (RT) of smaller tRNA fragments (like tRFs or tiRNAs) over full-length tRNAs. This is the case, because full-length tRNAs are (1) highly structured, and (2) also (more heavily) modified—in contrast to tRFs or tiRNAs—which will impede their RT required for NGS. Hence, the presence of tRFs, as observed in our study, is likely an overestimate in respect to their actual abundance in virus-infected cells, as evident by northern blot analysis.

However, by northern blot analysis, we were indeed able to confirm the presence of selected 5′-fragments for tRNA^Glu(TTC)^ and tRNA^Lys(TTT)^, respectively, which showed an increased abundance in host cells infected with both SARS-CoV-2 variants compared to uninfected cells (Figure 5G). Interestingly, we observed a decreased abundance of the 5′-tRF5^Glu(TTC)^ fragment in Delta-infected cells as compared to Omicron BA.2-infected and uninfected cells (Figure 5G, right). Therefore, tRNA^Glu(TTC)^ might be employed as a biomarker for disease severity and infectiousness in the future.

### Differentially expressed snoRNAs

**Figure 6:**
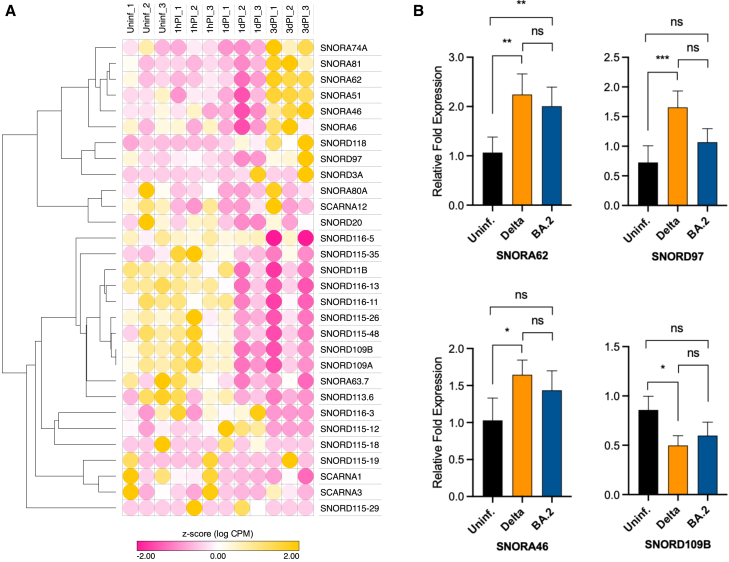
Differentially expressed snoRNAs in SARS-CoV-2-infected NHBE cells (A) Heatmap of significantly differentially expressed snoRNAs in uninfected (Uninf.) and SARS-CoV-2-infected following 1 h post-infection (1hPI), 24 h or 1 day post-infection (1dPI), and 72 h or 3 days post-infection (3dPI), indicated by *Z* score values. Positive values (yellow) represent high expression and negative values (pink) indicates low expression. (B) Validation of selected snoRNA candidates by RT-qPCR in (A) using Uninf. and SARS-CoV-2 (Delta or Omicron BA.2)-infected NHBE cells (3dPI). Bar graphs indicate mean ± standard deviation of fold change in expression of snoRNA candidates in Delta- and Omicron BA.2 (BA.2)-infected NHBE cells relative to Uninf. cells. The experiment was performed in triplicates using at least three independent samples (*n* = 3) for each group. Statistical analysis was carried out using Fisher’s LSD test. ∗*p* < 0.05, ∗∗*p* < 0.01, and ∗∗∗*p* < 0.001; and ns, not significant.

In addition to tRNAs, we also identified differentially expressed snoRNAs in SARS-CoV-2 Delta virus-infected epithelial cells. By employing the computational tools Manatee and exceRpt, a total of 30 dysregulated snoRNAs of the SNORD or SNORA class were found in Delta-infected NHBE cells (Figure 6A; Table S13). Here, the abundance of the majority of snoRNAs was found to be decreased (Figure 6A). Validation of selected candidate genes by RT-qPCR showed that the snoRNAs, whose expression was most differentially regulated in Delta-infected NHBE cells relative to the control, included SNORA46 (upregulated), SNORA62 (upregulated), SNORD97 (upregulated), and SNORD109B (downregulated) (Figure 6B). In contrast to expression changes induced by Delta infection, RT-qPCR validation of Omicron BA.2-infected cells only identified significant regulation of SNORA62, while expression of SNORA46, SNORD97, and SNORD109B was not significantly altered (Figure 6B). In addition to SNORA or SNORD snoRNAs, we also identified three Cajal body-specific RNAs from the snoRNA class, designated SCARNA1, SCARNA3, and SCARNA12, whose expression levels were found to be downregulated in Delta-infected NHBE cells (Figure 6A).

In contrast to canonical snoRNAs that target rRNAs, snRNAs, or tRNAs for modification, we also observed a reduced abundance of so-called “orphan” snoRNAs, i.e., SNORA51, SNORA80A, SNORA80, SNORD109A and B, and all gene copies of SNORD115 and SNORD116, as well as SNORD113 in SARS-CoV-2-infected cells. All of these orphan snoRNAs lack complementarity to canonical rRNA, snRNA, or tRNA targets. We previously identified SNORD108A and B, SNORD115, and SNORD116 to be located on the maternally imprinted chromosomal region 15q11-13, the Prader-Willi-Syndrome (PWS) gene locus.^39^ Thereby, SNORD116 has been shown to be directly involved in the etiology of PWS.^40^ In addition, SNORD113, whose expression was also found to be downregulated in SARS-CoV-2-infected NHBE cells, maps to a second maternally imprinted locus on chromosome 14.^41^

### Differentially expressed miscellaneous ncRNAs

The remaining ncRNAs, which are differentially expressed upon SARS-CoV-2 infection but cannot be grouped into larger RNA classes, were classified as miscellaneous RNAs (miscRNAs). The current literature makes a rather arbitrary distinction between small and lncRNA, based solely on the size of the ncRNAs, i.e., assigning the term small ncRNAs to RNA species smaller than 200 nt and long intergenic ncRNAs (lincRNAs) to ncRNAs longer than 200 nt, which is not based on their functions.

Interestingly, in addition to ncRNA species in the 20–200 nt size range from our study, we also identified lincRNAs sized >200 nt (similar to identification of mRNAs exhibiting sizes of >200 nt, which could represent ESTs). This can be explained either by the presence of smaller fragments of lincRNAs sized >200 nt (e.g., degradation fragments) or by RT library generation, where only partial sequences of these RNA species may be identified due to premature stops during RT (e.g., due to secondary structures or modifications of these RNAs).

To clarify which of the aforementioned scenarios applies, we performed northern blot analysis of selected dysregulated ncRNAs for which we obtained smaller sized sequence contigs, i.e., both for RNA species smaller or larger than 200 nt. For U2 snRNA, 7SL RNA, vtRNAs, or YRNAs, we showed that predominantly full-length RNAs were detected, while smaller RNA species were present only at very low levels, and their expression did not differ between uninfected and infected cells (Figure S4). One exception was tRNA fragments, the presence of which was also confirmed by northern blot analysis (Figure 5).

**Figure 7:**
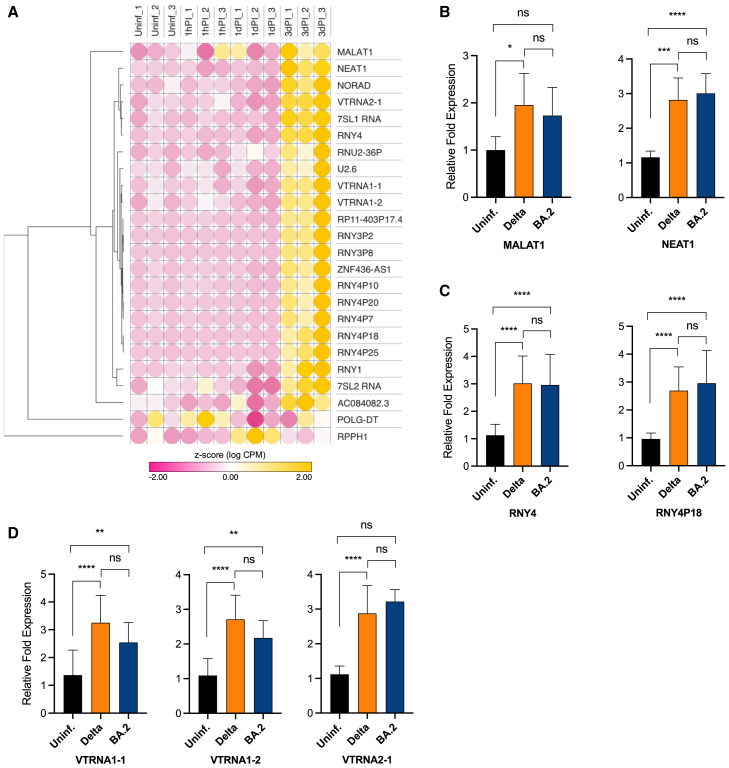
Differential expression of miscRNAs and lincRNAs identified in SARS-CoV-2-infected NHBE cells (A) Heatmap of significantly differentially expressed miscRNAs and lincRNAs indicated by *Z* score values. Positive values (yellow) indicate high expression and negative values (pink) represent low expression. (B–D) Validation of selected lincRNA, Y RNA, and vault RNA candidates in (A) by RT-qPCR using uninfected (Uninf.) and SARS-CoV-2 (Delta or Omicron BA.2)-infected NHBE cells (3dPI). The experiment was conducted in triplicates using at least three independent samples (*n* = 3) for each group. Bar graphs indicate mean ± standard deviation of fold change in expression of selected candidates in Delta- and Omicron BA.2 (BA.2)-infected NHBE cells relative to Uninf. cells. Normalized Ct values were used for statistical analysis using two-way ANOVA followed by Tukey’s post-hoc test. ∗*p* < 0.05, ∗∗*p* < 0.01, ∗∗∗*p* < 0.001, and ∗∗∗∗*p* < 0.0001; and ns, not significant.

Using the exceRpt data analysis, we observed a total of 101 differentially expressed miscRNAs in SARS-CoV-2-infected NHBE cells (1hPI, 1dPI, and 3dPI). Of these, the majority of candidate genes were composed of different Y RNA species, and the majority of ncRNAs showed a higher abundance in SARS-CoV-2 Delta-infected cells as compared to uninfected cells (Figure 7A; Tables S14 and S15). In contrast, the lincRNAs POLG-DT and the ribonuclease P RNA component H1 (RPPH1) showed different expression levels at different days of infection (i.e., at 1hPI, 1dPI, and 3dPI) compared to uninfected cells (Figure 7A). POLG-DT expression showed an oscillating expression pattern over time, in which it was elevated at the very onset of infection (1hPI), downregulated at 1dPI, and upregulated again at 3dPI, as compared to the untreated cells. Interestingly, POLG-DT has also been shown to be one of the most prominently downregulated lincRNAs in colorectal cancer.^42^ Similar to POLG-DT, RPPH1 also exhibited an oscillating expression pattern post infection, but reversed to that of POLG-DT, i.e., expression levels of RPPH1 in Delta-infected NHBE cells were upregulated at 1dPI but were decreased at 1hPI and 3dPI (Figure 7A). Thereby, RPPH1 encodes the RNase P moiety of the ribonucleoprotein particles (RNPs), which functions as an endoribonuclease that cleaves tRNA precursor molecules for processing of their leader sequence to generate mature 5′-ends.^43^

From the class of lincRNAs, the expression of metastasis-associated lung adenocarcinoma transcript 1 (*MALAT1*) and *NEAT1* RNAs was shown to be upregulated in Delta- and Omicron BA.2-infected NHBE cells (Figure 7B), although the upregulation in BA.2-infected cells did not reach statistical significance. The lincRNA NORAD, whose abundance is also increased in virus-infected cells, is expressed in endothelial cells and has a critical role in several cancers, thereby promoting cell proliferation.^44^ This also applies to the lincRNA *AC084082.3*, which is associated with increased risk of gallbladder cancer.^45^ Other lincRNAs with upregulated expression at 3dPI include *ZNF436-AS1*, an antisense lincRNA of unknown function, and *RP11-403P17.4*, which was previously described to be upregulated in mitochondrial myopathies.^46^

From the group of upregulated small ncRNAs (i.e., smaller than 200 nt), we identified Y RNA species as the most abundant differentially expressed ncRNAs in Delta-infected NHBE cells (Figures 7A–7C and Tables S14 and S15). Y RNAs belong to the class of small ncRNAs with a size of 84–113 nt and are highly conserved throughout evolution.^47^ They are classified into four distinct species: Y1, Y3, Y4, and Y5, with Y2 being a truncated form of Y1. Y RNAs form RNPs with two different proteins, Ro60 and La proteins.^48^ In our study, we observed that all Y RNA candidates (e.g., RNY3P2 and RNY4), which were mapped proportionally to the expression of uniquely mapping reads, were upregulated (Figure 7A; Table S14). Also, we identified multiple reads of Y RNA transcripts, which were mapped to more than one transcript/genomic location in the reference sequences (Table S15). From the selected candidates, we showed that RNY4 was found to be consistently induced in both Delta- and Omicron-infected NHBE cells, relative to uninfected cells (Figure 7C). In addition to Y RNAs, we also validated differential expression of three species of vtRNAs by RT-qPCR (i.e., VTRNA1-1, VTRNA1-2, and VTRNA2-1), which exhibited a higher expression in Delta- and Omicron BA.2-infected NHBE cells compared to uninfected cells (Figure 7D). Finally, we also observed an upregulation of 7SL RNA expression in Delta-infected NHBE cells. 7SL RNA represents the RNA component of the signal recognition particle (SRP) and is required for membrane trafficking of proteins destined to the endoplasmic reticulum or the cytoplasmic membrane.^49^

While the presence of smaller RNA species has previously been reported for some ncRNAs, such as 7SL, vtRNAs, or Y RNAs, in SARS-CoV-2-infected cells,^50^ we were unable to detect significant amounts of these fragments by northern blot analysis (Figure S4). Importantly, only after prolonged exposure of northern blots, small amounts of these ncRNA fragments were observed in our analyses. However, their low abundance and sizes did not differ between uninfected and virus-infected cells (Figure S4). The presence of smaller-sized fragments derived from *bona fide* ncRNAs or lincRNAs might therefore be explained by the application of an RNA-seq approach that depends on RT of RNAs, resulting in smaller sequence reads due to the structure or modification of RNAs. Thus, our study highlights the general requirement to validate the presence of RNA fragments derived from lncRNA, which are only predicted by RNA-seq analysis (for example, by northern blotting).

## Discussion

In this study, we set out to identify differentially expressed ncRNAs following infection of a 3D tissue model of human bronchial epithelial cells with the Delta variant of SARS-CoV-2. From differentially expressed ncRNAs identified in Delta-infected cells, we also investigated whether these were similarly differentially expressed in cells infected with the Omicron BA.2 variant. Various time points post infection were monitored to obtain a more detailed picture of the processes within host cells at the cellular barrier. While gene expression was similar to uninfected cells at 1 h and 1 day post infection with both virus variants, the number of differentially expressed genes was altered in virtually all RNA types on day 3 post infection. To date, only a few studies have investigated the regulation of cellular small RNAs,^50^^,^^51^ while the transcriptomic response of cells to viral infection has been investigated with respect to protein-coding RNAs or lncRNAs for several viruses, including SARS-CoV-2.^52^^,^^53^^,^^54^^,^^55^ Here, we performed small RNA-seq with library sizes of 20–200 nt, which allowed the detection of very short RNA types such as miRNAs (∼19–25 nt), piRNAs (∼25–32 nt), and also the longer snoRNAs (∼60–120 nt), tRNAs (∼70–100 nt) and their shorter fragments, as well as other classes of short ncRNAs such as Y RNAs (∼84–113 nt) and vtRNAs (∼80–150 nt).

In addition to short RNAs, our sequencing analysis also resulted in the alignment of reads to longer RNA transcripts, including both protein-coding RNAs and lncRNAs. Although the library preparation approach employed suggests that these might only constitute fragments of full mRNAs, the expression patterns of these mRNA fragments are consistent with the previously reported mRNAs regulated in SARS-CoV-2-associated mRNA sequencing studies.^56^^,^^57^ Therefore, our study is one of the first to illustrate comprehensive SARS-CoV-2-mediated changes in protein-coding as well as small RNA expression in human respiratory epithelial cells.

**Figure 8:**
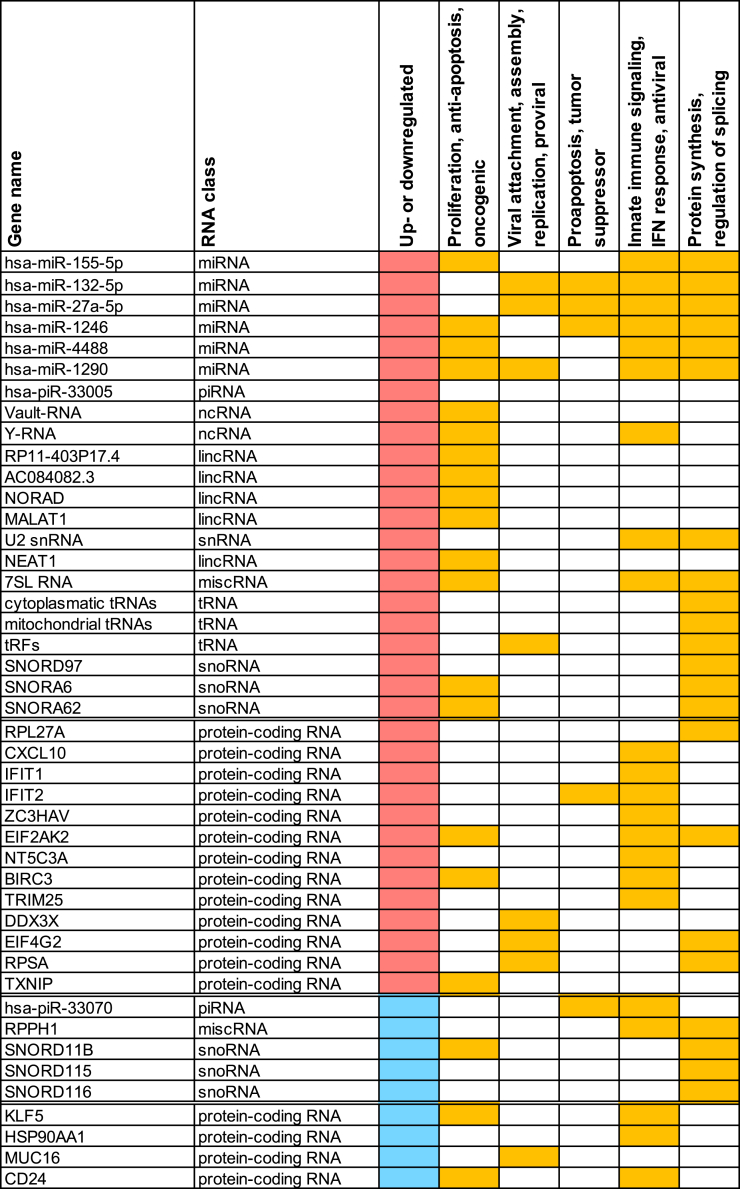
Involvement of differentially expressed RNA candidates with specific cellular pathways Allocation of upregulated (red) and downregulated (blue) protein-coding genes and non-coding RNA classes to the conserved pathways, namely “Proliferation, anti-apoptosis, oncogenic,” “Viral attachment, assembly, replication, proviral,” “Proapoptosis, tumor suppressor,” “Innate immune signaling, IFN response, antiviral,” and “Protein synthesis, regulation of splicing” based on a literature search of the respective genes and pathway terms.

Our study revealed 27 significantly dysregulated protein-coding genes associated with coronavirus disease, chemokine signaling pathway, and viral protein interaction, i.e., antiviral and pathogen recognition (Figure 8). To gain a more systematic view of the cellular processes involved, we assigned these significantly up- and downregulated protein-coding genes, as well as ncRNA genes, in SARS-CoV-2-exposed human airway epithelial cells to the following pathways (Figure 8).

### Innate immune signaling and type I IFN response

Here, we identified several components of the IFIT-CXCL10-EIF2AK2-ZC3HAV1 axis, a network of innate immune responses that is critical for host defense against viral pathogens such as SARS-CoV-2, which were significantly upregulated. Upon viral encounter, this axis involves induction of interferons, activation of antiviral proteins such as IFIT1 and IFIT2, inhibition of viral replication via EIF2AK2 and ZC3HAV1, and degradation of viral RNA by recruitment of the cellular RNA degradation machinery to viral RNA molecules by ZC3HAV1. In particular, we demonstrated a significant upregulation of the IFIT1/2-CXCL10 axis upon infection of the highly differentiated pseudostratified primary human respiratory epithelium with SARS-CoV-2 Delta on day 3 post infection. This may be related to the initiation of excessive innate and adaptive immune responses, as observed particularly during the first waves of the COVID-19 pandemic. At this time, more virulent wild-type and Delta variants were circulating,^58^ which may also result in extensive recruitment of lymphocytes to sites of infection when prolonged activation occurs.

We also identified differential expression of the genes *TRIM25*, *KLF5*, and *RPSA*, which encode important regulators of antiviral immune responses and are interconnected to the aforementioned axis. TRIM25 is primarily involved in the early detection of viruses and initiates antiviral signaling pathways, whereas KLF5 modulates expression of immune-related genes and inflammation and has recently been highlighted as an antiviral factor.^59^ While *TRIM25* was again significantly upregulated by both virus variants, which might lead to extended periods of type I IFN responses, *KLF5* expression was downregulated, which could weaken antiviral immunity against SARS-CoV-2. In addition, we observed an increased abundance of *RPSA*, a ribosomal protein with several functions described in the following text. Functional screening also identified RPSA as a nuclear protein that recognizes viral nucleic acids and predominantly promotes proinflammatory cytokine gene expression in antiviral innate immunity. Hence, RPSA may represent a potential novel target for combating SARS-CoV-2 infection.^60^

In addition to protein-coding genes, we specifically aimed to identify differentially expressed host cell ncRNA species between 20 and 200 nt in size that would either promote viral replication and thus likely would be induced by SARS-CoV-2, as well as ncRNAs that the host cell might use as a defense mechanism to prevent viral replication. We also anticipated to identify differentially expressed ncRNAs involved in both pathways. Furthermore, we investigated whether differential expression of ncRNAs would be conserved between the two SARS-CoV-2 variants, i.e., the Delta and the BA.2 Omicron strains.

As one of the most prominently upregulated and abundant ncRNAs in our screen, we identified three out of four Y RNA species (i.e., various gene copies of Y RNA1, 3 and 4, respectively) that have previously been shown to be dysregulated during viral infection.^61^ It has also been suggested that Y RNAs, as observed for aforementioned protein-coding genes, may elicit antiviral immune responses by activating pattern recognition receptors (PRRs) such as Toll-like receptor 7^48^ and retinoic acid-inducible gene I (RIG-I)-like.^61^ In particular, increased expression of RNY4 has been observed in dengue virus, measles, and HIV-1 infections. In addition to RNY4, other Y RNA family species act as ligands leading to activation of the RIG-1 signaling pathway, as observed for 7SL and RPPH1 RNAs.^61^ PRRs recognize viral genetic material at the onset of viral infection and their activation leads to induction of the IFN signaling pathway.^61^ This is consistent with the analysis of differential expression of protein-coding genes, in which we also observed an upregulation of IFN-stimulated protein-coding genes such as *CXCL10*, *IFIT1*, *IFIT2*, *ZC3HAV1*, and *EIF2AK2* (Figure 8, “Innate immune signaling, IFN response, antiviral”; Figures 2B and 2C; Table S1), consistent with the upregulated expression of Y RNAs (Figures 7A–7C; Tables S14 and S15). Thus, our findings suggest a potential role for Y RNAs in the regulation of antiviral immune responses.

As observed for Y RNAs, we found an increased abundance of 7SL RNA at day 3 post viral infection. 7SL RNA, the RNA component of the SRP, is required for protein secretion or protein insertion into the cellular membrane.^62^ It has previously been reported that cytoplasmic 7SL RNA can act as a direct ligand of RIG-1 and thereby regulate IFN signaling in cells lacking NSUN2, an m^5^C methyltransferase. Inhibition of 7SL RNA expression in NSUN2-depleted cells attenuates IFN signaling, thereby partially rescuing viral replication and gene expression.^63^ During viral infection, as has been demonstrated for U1 and U2 snRNAs, 7SL RNA is targeted by the SARS-CoV-2 proteins NSP8 and 9, respectively, thereby interfering with secretion of cytokines and other cellular immunomodulatory proteins by the SRP.^64^ Hence, the increased expression of 7SL RNA, as observed for U2 snRNA, may resemble a host cell defense mechanism to counteract binding of the viral proteins NSP8 and 9 to the 7SL RNP.

In addition to 7SL RNA, the RNA component of the nuclear RNaseP endonuclease, i.e., RPPH1, was also found to be upregulated at 2dPI, but downregulated at 3dPI. As shown for 7SL RNA, RPPH1 can act as a direct ligand for RIG-1 and its increased expression in the cytoplasm induces the RIG-1-mediated IFN response during viral infection. Knockdown of RPPH1 and 7SL RNA attenuates IFN signaling and also partially rescues viral replication.^63^ In contrast, overexpression of RPPH1 promotes inflammation and cell proliferation through the Gal-3/Mek/Erk signaling pathway in mesangial cells.^65^ Thus, downregulation of RPPH1 at 3dPI may be a mechanism by which SARS-CoV-2 is able to regulate both inflammation and cell proliferation.

### Viral attachment, replication, and assembly

In contrast to the protein-coding genes mentioned previously, the abundance of *MUC16*, *KLF5*, *EPM2AIP1*, *HSP90AA1*, *FAM204A*, and *CD24* was found to be considerably reduced in SARS-CoV-2-infected cells (Figure 2). However, when overexpressed, MUC16 has been shown to be protective against SARS-CoV-2 in organoid cultures.^66^ Our data indicate that the virus is able to reduce MUC16 expression in primary respiratory cells, thereby facilitating interactions with the ACE2 receptor as an entry point.

In contrast to *MUC16*, we found that the ribosomal protein *RPSA*, a ribosomal protein of the large ribosomal subunit, was upregulated at 3dPI. Interestingly, RPSA has previously been described to have several functions: (1) as a protein interacting with the ribosomal protein L27A and (2) by being a precursor for the 67 kDA Laminin Receptor (67LR), which gains laminin-binding capability due to post-translational modifications. By the latter function, it has been reported to serve as a membrane receptor for many pathogenic bacteria and viruses.^67^^,^^68^ Recently, Jiang et al. reported that nucleus-localized RPSA can also recognize viral nucleic acids (both herpes simplex virus 1 [HSV-1] genome DNA and influenza A virus genome RNA) within the host nucleus, followed by induction of pro-inflammatory cytokine production via epigenetic modifications.^60^ These data highlight an additional role for RPSA in the control of viral infection-triggered innate inflammatory responses. Notably, at 3dPI we also found an increased abundance of *RPL27A* in SARS-CoV-2-infected cells, possibly indicating a role for RPSA-RPL27A interaction in viral attachment and replication. Both proteins have also been reported to be upregulated in various cancers.^69^^,^^70^ In the future, it will be interesting to investigate whether all these functions of RPSA are interconnected and to investigate the underlying mechanism in viral infection and propagation. Thus, RPSA could be employed as a novel biomarker and target to combat SARS-CoV-2 infection.

DDX3X and BIRC3 are involved in cellular immune responses to SARS-CoV-2 infection. While DDX3X has been shown to promote viral replication, BIRC3 is more likely to be involved in the production of pro-inflammatory cytokines. Several animal studies have described a lower intrinsic virulence of Omicron variants compared to the Delta variant, with milder disease manifestations and reduced inflammatory responses.^71^^,^^72^ Consistent with these findings, in our respiratory model, we demonstrated an upregulation of the aforementioned genes involved in the mimicking of the “cytokine storm” phenomenon during Delta infection.

As observed for protein-coding genes, specific miRNAs are also known to affect RNA virus replication and pathogenesis as they can bind directly to RNA virus genomes at miRNA binding sites both in non-translated regions and coding regions of viral proteins.^73^ Interestingly, the effects of miRNA regulation on viral processes can be diverse. For example, miRNA miR-155, which showed the highest upregulation in our screen, is also upregulated in the vast majority of viral infections.^74^ It has been shown that miR-155 promotes antiviral immunity in hepatitis B virus (HBV) infection via the SOCS1 and JAK/STAT pathways.^75^ Furthermore, induction of miR-155 expression *in vitro* reduces the viral load during HBV infection.^76^ In line with this observation, upregulation of miR-27a inhibits adenovirus infection via target gene interactions, which is counteracted *in vivo* by small ncRNAs encoded by the viral genome.^77^ Taken together, this suggests an antiviral role for the aforementioned miRNAs, including during SARS-CoV-2 infection.

On the other hand, increased expression of miR-1290, also identified in our study, has been shown to promote viral replication during influenza A virus infection, while the application of a miR-1290 antagonist was found to reduce viral protein levels and viral titers.^78^ In addition, miR-132, which was upregulated in our screen, has been shown to facilitate viral replication during infection with Kaposi’s sarcoma-associated herpes virus, HSV-1, and human cytomegalovirus by inhibiting the expression of p300, which is known to be essential for the initiation of antiviral immunity.^79^ Thus, by being upregulated in our screen, the induction of these miRNAs appears to be employed by SARS-CoV-2 to promote viral infection.

For downregulated expression of miRNAs in our screen, it has been shown that application of a miR-181 mimic limits the replication of porcine reproductive and respiratory syndrome virus.^80^ The miRNA miR-874, which is downregulated in various cancers and non-cancer diseases, regulates apoptosis via a variety of target genes and pathways,^81^ and application of a miR-874 mimic decreases enterovirus 71-induced apoptosis,^82^ also suggesting downregulation of these miRNAs by SARS-CoV-2 infection as a proviral mechanism. Interestingly, the primate-specific miR-576 sensitizes cells to viral replication, whereas inhibition of miR-576 prevents infection.^83^ Thus, regardless of the direction of regulation, individual miRNAs may serve anti- and proviral processes and pathways, depending on their respective target genes.

### Anti-inflammatory signaling

Based on an integrative analysis of a genome-wide CRISPR dropout screen and a genome-wide association study analysis between variants of identified host factors and clinical features, Hou et al. identified KLF5 as a significant anti-viral factor, with genetic variations linked to COVID-19 hospitalization and severe symptoms, as well as a downregulation of *KLF5* mRNA expression in severe COVID-19 cases compared to mild cases, thereby highlighting its clinical relevance in the disease.^59^ This is consistent with our data, where we detected a significant downregulation of this factor in Delta, but not Omicron BA.2 infection, which is associated with a milder disease progression.

Soluble CD24 (CD24Fc) has been identified as an effective agent in mitigating extensive inflammatory response triggered by damage-associated molecular patterns by binding to extracellular high-mobility group box 1 and heat shock proteins (HSPs). Administration of CD24 can rapidly reduce systemic inflammation and re-establish immune homeostasis in COVID-19 patients, underscoring its potential as a novel therapeutic for severe COVID-19.^84^ Given its anti-inflammatory properties, it is not surprising that our study detected a significant downregulation of *CD24* as well as *HSP90AA1*, particularly by the Delta variant. This downregulation likely represents a viral strategy to evade the broad suppression of virus-mediated inflammatory responses mediated by these mechanisms.

### Gene regulation and protein synthesis

Among the protein-coding genes involved in the translation of SARS-CoV-2 mRNAs, we identified upregulated expression of elongation factor *EIF4G2*. EIF4G2 shares similarity with the C-terminal region of eIF4G.^85^ However, unlike eIF4G, which supports both cap-dependent and -independent translation, EIF4G2 functions as a general repressor of canonical translation by forming translationally inactive complexes and initiating only at non-AUG codons. Interestingly, it has previously been shown that a significant portion of SARS-CoV-2 open reading frames initiate from a CUG rather than an AUG codon.^86^

Consistent with the regulation of cellular protein synthesis by SARS-CoV-2, we also identified an increased abundance of a significant number of nuclear and mitochondrial tRNAs (Figure 5), which might promote viral protein synthesis. However, host defense mechanisms such as the synthesis of interferons and cytokines might also be increased. Emerging data show that viruses can accomplish this by either adjusting the host cell tRNA pool to better match viral codon usage or altering and optimizing viral codon usage toward the codon repertoire of the host.^87^ For the latter scenario, it was indeed recently shown by Dilucca et al.^88^ that SARS-CoV-2 adopts codon usage of its own genes to the human host codon usage for optimal translation.

In respect to host cell defense mechanisms, as for upregulated cytoplasmic tRNAs from our screen, e.g., tRNA^Sec^, this might reflect a defense mechanism to enhance translation of selenocysteine containing proteins, such as glutathione peroxidase (GPx). Interestingly, the GPx family is essential for maintaining cellular redox balance and countering the overproduction of reactive oxygen species, which has been linked to viral infections.^89^

Interestingly, in a recent study on chronic kidney disease, we identified exosomal mitochondrial tRNAs as biomarkers of renal inflammation.^90^ In addition to full-length tRNAs, we also identified increased abundance of some tRNA halves from SARS-CoV-2-infected cells, which have been implicated in response to cellular stress conditions such as oxidative stress or viral infection.^87^ Importantly, sustained activation of stress responses can lead to inflammation and disease pathogenesis.^91^ Previously, it was demonstrated that during infection, the respiratory syncytial virus shows an increased abundance of the lysine and glycine 5′-tRNA halves, i.e., 5′-tRF^Lys(CCC/TTT)^ and 5′-tRF^Gly(GCC/CAC)^, which promotes virus propagation.^92^ Interestingly, we also found a similar increase of 5′-tRF^Lys(TTT)^ halves, by both SARS-CoV-2 variants at 3dPI (Figures 5E and 5F; Table S12).

In addition to tRNAs and tRFs, we identified a number of differentially expressed canonical snoRNAs whose expression was increased upon SARS-CoV-2 infection. snoRNAs are classified as SNORD or SNORA RNAs based on conserved sequence and structure motifs.^93^ Thereby, the canonical snoRNAs identified in our study were involved in protein synthesis by modifying ribosomal RNAs, i.e., 18S and 28S rRNAs, but have also been reported to be involved in splicing by modification of spliceosomal RNAs, i.e., snRNAs. Hence, their observed differential expression might be correlated to alterations in splicing patterns and/or changes in protein synthesis. Notably, differences in the expression of snoRNAs have previously been reported to correlate with the severity of SARS-CoV-2 infection.^94^

In contrast to the canonical snoRNAs described previously, which target rRNAs, SNORD97, located within an intron of the *EIF4G2* gene, modifies tRNA^Met^ at position C34 within its anticodon loop for 2′-O-methylation.^95^ Vitali and Kiss demonstrated that cleavage of tRNA^Met^ within its anticodon loop by the stress-induced endonuclease angiogenin is inhibited by 2′-O-methylation.^95^ Hence, increased expression of SNORD97 may indeed prevent tRNA^Met^ cleavage, thereby promoting protein synthesis and cell survival. In addition to miRNAs, snoRNAs have also been reported to be involved in inflammatory processes during viral infections including HSV-1.^96^

In eukaryotic genomes, including humans, snoRNAs are mostly encoded within introns of protein-coding genes and subsequently processed by splicing. Therefore, an increased or decreased abundance of snoRNAs in SARS-CoV-2-infected cells could either be functionally linked to the snoRNA itself or, alternatively, correlated with the expression of the host gene (Table S16)^97^ encoding the respective snoRNA, or both. Thus, the differential expression of snoRNAs observed in SARS-CoV-2-infected cells might serve as a sign of differential expression of their host genes.

Thereby, up- or downregulation of snoRNAs always correlated with the concomitant up- or downregulation of host genes identified in our screen (Table S16). This is observed for the SNORD97 host gene (EIF4G2, see previous text), as well as for the SNORA6 and SNORA62 host gene (RPSA, see previous text). As for the aforementioned snoRNAs, SNORA46 is located within the *AHSA1* host gene. AHSA1 has previously been reported to directly interact with HSP HSP90AA1,^98^ also identified in our screen (see previous text) which has been demonstrated to facilitate SARS-CoV-2 virion assembly. In addition to *AHSA1*, other host genes of upregulated snoRNAs reported to be involved in general immune response^98^ might also impact viral infection and propagation, such as *MATR3* (innate immune response), RFWD2 (induction of cell proliferation), or *PHB2* (involved in RIG-1 mediated signal transduction) (see Table S16).

### Cell proliferation, anti-apoptosis, and oncogenesis

Infection by pathogens, such as viruses, requires that human host cells do not undergo apoptosis prior to viral replication, assembly, and propagation. Also, virus infection can induce oncogenesis by activating the inflammatory response to stimulate the growth of infected cells and inhibit apoptosis. An aberrantly activated inflammatory response correlated with disease severity in COVID-19 patients.^60^

Protein-coding genes *EIF2AK2*, *BIRC3*, and *TXNIP*, previously described to be involved in the regulation of apoptosis and cell proliferation upon viral infection,^99^^,^^100^^,^^101^^,^^102^ were also upregulated in our study. Moreover, the increased expression of several lincRNAs at 3dPI is consistent with previously described functions in promoting cell proliferation and oncogenesis, thereby inhibiting apoptosis. These include the lincRNAs *NEAT1* (nuclear enriched abundant transcript 1 or nuclear paraspeckle assembly transcript 1) and *MALAT1* (metastasis-associated lung adenocarcinoma transcript 1). *NEAT1* is a lncRNA located on chromosome 11 that is widely expressed in mammalian cell types and, similar to our data, was found to be overexpressed in several inflammation-related disorders^103^ (Figures 7A–7C). In addition, NEAT1 has been shown to be an essential paraspeckle component in hypoxia, thereby serving as a key translational regulator, and is active at internal ribosome entry sites of (lymph-)angiogenic and cardioprotective factor mRNAs.^104^ *MALAT1* , also known as NEAT2 (nuclear enriched abundant transcript 2), is a large, infrequently spliced ncRNA that is conserved across the mammalian lineage.^105^ Its dysregulated expression in many types of cancerous tissues is associated with abnormal cell proliferation, invasion, and metastasis of tumor cells.^106^ Promotion of cell proliferation and thus inhibition of apoptosis also applies to the lincRNAs NORAD (*LINC00657*) and *AC084082.3*. Kyoto Encyclopedia of Genes and Genomes (KEGG) pathway analysis of lincRNA *RP11-403P17.4* has indicated its potential role in infectious diseases and regulation of the immune system, translation, signal transduction, and cell communication.^46^

In addition to lincRNAs sized >200 nt, also smaller miscRNAs such as vtRNAs, which have been reported to be involved in the inhibition of apoptosis, were found to be significantly upregulated in both Delta- and Omicron-infected cells. vtRNAs are between 80 and 150 nt in size and four different vtRNAs (i.e., VTRNA1-1, VTRNA1-2, VTRNA1-3, and VTRNA2-1) have been identified in humans. In a previous study, we also found that vtRNAs are upregulated in EBV-infected cells.^107^ vtRNAs have been described to participate in the inhibition of autophagy and apoptosis by directly binding to the autophagy receptor Sequestosome-1/p62 in human and murine cells.^108^ Thereby, vtRNAs regulate selective autophagy by binding p62 and interfering with oligomerization, a critical step of p62 function.^109^ Accordingly, an increased abundance of vtRNAs during viral infection would benefit viral replication by inhibiting apoptosis of infected cells.

Activation of autophagy can antagonize viral infections by delivering cytoplasmic viral components to lysosomes for degradation, promoting inflammatory response, antigen presentation, and clearance for pathogen recognition.^110^ While Horos et al. showed that EBV can evade autophagy to promote viral infection,^109^ Durgan et al. indicate that influenza A virus could trigger autophagy for its benefit via autophagy-related 8-phosphatidylserine (ATG8-PS) alternative lipidation mechanism,^111^ indicating that viruses can wield a double-edge sword to circumvent host immunity mechanisms.

From the class of small ncRNAs, we identified upregulated expression of Y RNA 1, 3, and 4, which have also been described to be involved in RIG-1 signaling.^61^ In addition, Y RNAs have been reported to act as scaffolds for several proteins (e.g., Ro60, Argonaute and La protein, respectively) that regulate cellular processes such as DNA replication, RNA processing and stability, thereby promoting cell proliferation and cellular stress responses.^48^^,^^61^

Also, the involvement of dysregulated miRNAs in regulation of apoptosis mechanisms has been shown. Upregulation of the hypoxia-associated miRNA miR-210,^112^^,^^113^ as well as miR-155^114^^,^^115^ and miR-1290,^116^ showed anti-apoptotic properties. On the contrary, miR-132^117^ and miR-4488,^118^ which were both also upregulated in our screen, were shown to promote apoptosis. The miRNAs miR-874 and miR-110, whose expression was downregulated in our study, are known to inhibit cell proliferation and induce apoptosis, while miR-181a-3p has been reported to suppress apoptosis.^119^ Among miRNAs whose expression was validated by qPCR, only hsa-miR-155-5p showed higher expression in Delta-infected cells compared to Omicron BA.2-infected cells in our 3D tissue model. Interestingly, miR-155 upregulation has been specifically associated with the induction of apoptosis, thereby exerting antiviral effects in response to viral infection.^74^ This bidirectional regulation of miRNAs associated with apoptosis pathways appears to serve multiple purposes, balancing between both pro- and antiviral effects and highlighting the critical role of miRNAs in fine-tuning the host response to viral infection.

Lastly, piRNAs have also been implicated in the regulation of cell proliferation and apoptotic pathways, with both pro- and anti-apoptotic effects reported.^33^^,^^120^ One of the few curated databases for piRNAs is piRNAdb, which also includes predicted (mRNA or lincRNA) targets of piRNAs, is based on piRNA sequences that overlap with protein-coding and non-coding regions of transcripts.^34^ While no target information was available for the majority of dysregulated piRNAs, a number of interesting piRNA::mRNA associations emerged (Table S10). For example, hsa-piR-32157 showed a number of putative targets involved in suppression of apoptosis and antiviral response (i.e., *IFT88*, *CRTC1*, *FAM83C*, *HAUS8*, *LINC01132*, *OTUB2*, and *PPM1K-DT*). Interestingly, while both IFT88 (intraflagellar transport 88) and OTUB2 (OTU deubiquitinase, ubiquitin aldehyde binding 2) are involved in suppression of apoptosis,^121^^,^^122^ HAUS8 (HAUS augmin like complex subunit 8) has been shown to regulate antiviral signaling.^123^ hsa-piR-32186 targets the transcription factor *DPF2* (double PHD fingers 2, also known as BAF45D), whose knockdown reduces cell viability and induces apoptosis.^124^ The genes *SHF* (Src homology domain containing F) and *HTT* (huntingtin), which are known to inhibit apoptosis,^125^^,^^126^ are targeted by hsa-piR-28060 and hsa-piR-33041, respectively. Finally, hsa-piR-27429 is supposed to target *BIRC5* (baculoviral IAP repeat containing 5, also known as survivin), a protein of the intrinsic apoptotic pathway that inhibits apoptosis and promotes cell proliferation.^127^

## Conclusions

In this study, we aimed to decipher changes in the small RNA transcriptome upon infection with the highly pathogenic SARS-CoV-2 Delta variant. By RT-qPCR and northern blot analysis, we subsequently also investigated whether differential gene expression of the small RNAs could also be found in a second SARS-CoV-2 variant, the Omicron BA.2 strain. Indeed, with few exceptions, we could observe a similar differential expression of ncRNAs or mRNAs in both strains.

Compared to previously published reports,^128^^,^^129^ our study stands out by employing a primary human 3D tissue model representing the bronchial environment and by linking changes in the small RNA transcriptome caused by Delta or Omicron to five major pathways. Thereby, the most upregulated protein-coding genes were involved in (1) “innate immune signaling and type I IFN responses”; concomitant with this cluster, Y RNA, 7SL, and RPPH1, associated with RIG-1 signaling, were also differentially modulated by SARS-CoV-2 variants Delta and Omicron BA.2 within the respiratory epithelium. Other pathways differentially modulated by Delta or Omicron variants were (2) “viral attachment, assembly, and replication,” where downregulation of the extracellular matrix protein MUC16 might facilitate virus entry, while upregulation of RPSA-RPL27A interacting proteins might also favor viral attachment and replication; (3) “anti-inflammatory signaling,” which was associated with downregulation of *KLF5*, *CD24*, and *HSP90AA1*; and (4) “gene regulation and protein synthesis,” where *RPL27A*, *ZC3HAV*, *EIF4G2*, and *RPSA* were highly upregulated, and which coincided with increased expression of miRNAs, 7SL RNA, tRNAs and snoRNAs. Finally, a large cluster, especially in terms of small RNA regulation upon infection, was (5) “cell proliferation, anti-apoptosis, and oncogenesis,” where *EIF2AK2*, *BIRC3*, and *TXNIP* were significantly upregulated upon infection (Figure 8).

Interestingly, differences in gene expression between Delta and Omicron BA.2 were mainly observed for protein-coding genes such as *CXCL10*, *IFIT2*, and *ZC3HAV1*, with moderate differences in ncRNA expression between the two variants. Notably, hsa-miR-155-5p showed a significantly lower expression in BA.2-infected cells compared to the Delta variant, which may contribute to the less severe phenotype observed in patients. Conversely, the lower abundance of the 5′-tRF^Glu(TTC)^ fragment in Delta-infected cells compared to Omicron BA.2-infected cells might also correlate with disease severity. The remaining ncRNA responses did not significantly differ between the two variants, suggesting conserved host cell responses to SARS-CoV-2 infection.

While these differences in gene expression between Delta- and Omicron BA.2-infected cells were observed, one limitation of our study is that only a selected set of genes was directly compared between the two variants. As a result, RNAs that are differentially expressed only in response to Omicron BA.2 infection, but not in Delta-infected cells, could not be detected. Future studies should aim to address this gap by expanding the comparison to include a broader range of genes and exploring variant-specific responses in more detail.

Differentially expressed protein-coding genes and ncRNAs might help to identify novel biomarkers and targets to combat future SARS-CoV-2 infections. Under cellular stress, many ncRNAs, particularly tRNAs/tRFs, snoRNAs, and miRNAs, are secreted from host cells within exosomes, which can be used for the diagnosis of inflammatory diseases.^90^^,^^130^ Exosomal profiling of ncRNAs could thus enable risk stratification and therapy for high-risk SARS-CoV-2 patients (Tables S4, S9, and S11–S15).

Employing a human respiratory barrier model infected with Delta or Omicron BA.2 and an integrated approach that included not only small RNA preparation and NGS but also verification of the most up- or downregulated gene candidates by RT-qPCR or northern blot analysis, we were able to demonstrate the involvement of protein-coding genes and small RNAs in five major pathways of infectious processes. Understanding small ncRNA gene expression changes during viral infection will therefore facilitate knowledge of which classes of small ncRNAs are suitable for novel therapies to overcome viral replication and to support innate immune responses after virus entry. Although currently none of the circulating SARS-CoV-2 variants are classified as “variants of concern,” this research will help to combat future viral diseases by providing potential diagnostic and therapeutic avenues. The therapeutic potential of small RNA molecules is evident, with several siRNA therapeutics already approved by the FDA and EMA, and miRNA-based drugs showing promise in clinical trials.^131^

## Materials and methods

### Ethics statement

Written informed consent was obtained from all donors of leftover nasopharyngeal/oropharyngeal specimens and EDTA blood by the participating clinics. The Ethics Committee of the Medical University of Innsbruck (ECS1166/2020) approved the use of anonymized leftover specimens of COVID-19 patients for scientific purposes.

### NHBE cell culture

NHBE cells (NHBE, Lonza, cat# CC-2540 S, upper respiratory tract) are available in our laboratory and routinely cultured in ALI as described.^6^^,^^16^^,^^132^ Briefly, cells were cultured as a monolayer for 2–4 days until they reached 80% confluence. Cells were detached and seeded onto GrowDexT (UPM)-coated 0.33 cm^2^ porous (0.4 μm) polyester membrane inserts with a seeding density of 1 × 10^5^ cells per Transwell (Costar, Corning, NY, NY, USA). The cells were grown to near confluence in submerged culture for 3 days in specific epithelial cell growth medium according to the manufacturer’s instructions (Stemcell). Cultures were maintained in a humidified atmosphere with 5% CO_2_ at 37°C and then transferred to ALI culture for another four weeks until cells were fully differentiated. The epithelium was expanded and differentiated using airway media from Stemcell. The number of days in development was designated relative to initiation of ALI culture, corresponding to day 0.

### Vero cells

VeroE6/TMPRSS2/ACE2 is an engineered VeroE6 cell line expressing high levels of TMPRSS2 and ACE2 and highly susceptible to SARS-CoV-2 infection. This cell line was used to expand characterized Delta and Omicron BA.2 virus variants from patient isolates and to perform plaque assays to test infectivity. The cell line was obtained via the Centre for AIDS Reagents (CFAR, NIBSC) and is described in a study by Matsuyama et al.^132^

### Staining and high content screening

To visualize SARS-CoV-2 infection in 3D tissue models, cells were infected with clinical specimen of SARS-CoV-2 Delta and Omicron BA.2 variants and analyzed for characteristic markers in infection experiments on 3dPI. After SARS-CoV-2 exposure, 3D cell cultures were fixed with 4% paraformaldehyde. Intracellular staining was performed using 1× Intracellular Staining Permeabilization Wash Buffer (10×; BioLegend, San Diego, CA, USA). Cells were stained using acetylated tubulin-Alexa647 (Thermo Fisher Scientific, Waltham, MA, USA), and nuclei were stained using Hoechst (Cell Signaling Technologies, Danvers, MA, USA). Intracellular SARS-CoV-2 was detected using Alexa 594-labeled SARS-CoV-2 antibodies against N (Sino Biological, Beijing, China). The Alexa 594-labeling kit was purchased from Abcam, Cambridge, UK. After staining, 3D cultures were mounted in Mowiol. Images were analyzed using the Operetta CLS System (PerkinElmer, Waltham, MA, USA) and Harmony Software.

### Viral plaque assay

VeroE6/TMPRSS2/ACE2 cells (1.2 × 10^5^) were seeded in a 24-well plate with culture medium (DMEM high glucose medium supplemented with 10% fetal calf serum, 1% L-glutamine, and 1% penicillin/streptomycin; all reagents were obtained from Sigma Aldrich, Missouri, USA) and incubated overnight at 37°C and 5% CO_2_. On the following day, supernatants from uninfected, Delta-, and BA.2-infected NHBE cells were serial-diluted from 1:100 to 1:10,000, and VeroE6/TMPRSS2 cells inoculated for 1 h at 37°C and 5% CO_2_. After incubation, the inoculate was replaced with culture medium containing 1.5% carboxymethylcellulose (Sigma Aldrich). Cells were incubated for 3 days at 37°C and 5% CO_2_ before plaque visualization and counting. For this, cells were washed and fixed with 10% neutral buffered formaline (Sigma Aldrich) for 1 h at room temperature. Fixation was followed by staining using 0.5% (w/v) Crystal violet solution (Sigma Aldrich) for 15 min at room temperature.^16^

### Real-time RT-qPCR for absolute quantification of SARS-CoV-2

SARS-CoV-2 RNA was extracted using FavorPrep Viral RNA Mini Kit, according to manufacturer’s instructions (Favorgen Europe, cat no. FAVRE 96004, Austria). Sequences specific to 2 distinct regions of the nucleocapsid (N) gene, N1 and N2, and for the detection of a human housekeeping gene, ribonuclease P, were used. Single target assays of all 3 targets were performed in combination with the Luna Universal Probe One-Step RT-qPCR Kit (New England Biolabs, cat no. E3006, Germany). For absolute quantification using the standard curve method, SARS-CoV-2 RNA was obtained as a PCR standard control from the National Institute for Biological Standards and Control, UK. All runs were performed on a Bio-Rad CFX 96 instrument and analyzed by the Bio-Rad CFX Maestro 1.1 software (Bio-Rad, Germany).

### Viruses

Clinical specimens for SARS-CoV-2 variant Delta and Omicron subvariant BA.2 from sequenced COVID-19 positive swabs were propagated and subsequently used to infect cells at an MOI of 0.01. All experiments with live virus strains (infection and plaque assays) were performed under BSL3 conditions.

### Statistical analysis

Statistical analysis of differences in infection levels or cytokine production was performed utilizing the GraphPad prism software and using one-way ANOVA followed by Tukey’s post-hoc test.

### Total RNA isolation from SARS-CoV-2-infected NHBE cells

Total RNA of NHBE cells uninfected or infected with SARS-CoV-2 variants for 1 h, 24 h, and 72 h was extracted using miRNeasy extraction kit (QIAGEN) and according to the manual’s instructions. Briefly, cells were pre-washed with 1× PBS, lysed in Qiazol lysis solution and homogenized. Cell lysates were added with chloroform and centrifuged to separate the phases. The aqueous phase was transferred into a new Eppendorf tube, added with absolute ethanol, and subsequently transferred into a spin column. Following centrifugation, samples were washed with wash buffers and 80% of ethanol. Total RNA was eluted with nuclease-free water, quantified in Nanodrop (ND-1000, Avantor, VWR), analyzed in Bioanalyzer (Agilent), and stored at −80°C.

### Small RNA library preparation and sequencing

Total RNA extracts from NHBE cells were analyzed for RNA-sequencing, employing small RNA-seq library prep kit (Lexogen GmbH, Vienna, Austria) and Illumina-based sequencing (GENEWIZ, Azenta Life Sciences, Germany). A total amount of 500 ng of total RNA for each sample was used as a starting material for the cDNA library preparation. Reverse transcription (RT) was carried out as described in the kit manufacturer’s instructions. Briefly, input RNA was ligated with 3′ adapter, followed by removal of excess 3′ adapter through column purification and then ligation of 5′ adapter. Following conversion of RNA into cDNA, the library product was cleaned up and concentrated. Removal of linker-linker artifacts or separation of small RNA library from the total library was performed using magnetic beads purification. Final libraries were multiplexed and sequenced with Illumina NovaSeq technology, using 150-bp paired-end settings and targeting an average of 20 million read pairs per library. Raw reads were collected and pre-processed, generating FASTQ files for each sample.

### RNA-seq data analysis

The quality of sequencing reads was assessed using FastQC and MultiQC. Several analysis pipelines have been applied including exceRpt,^18^ Manatee,^21^ miRDeep2,^19^ and tRAX.^20^

Based on adapter trimmed.fastq files using skewer (v.0.2.2), the exceRpt small-RNA-seq pipeline (https://github.com/gersteinlab/exceRpt) was applied, including mapping onto the human genome (GRCh38), annotation and separate quantitation for different RNA classes such as miRNAs (miRBase), tRNAs (gtRNAdb), piRNAs (piRNAbank), and longRNAs (Genecode v.24 human annotation), and normalized to library size.

For applying the Manatee pipeline (https://github.com/jehandzlik/Manatee), adapters were trimmed using Fastp, sequencing pairs were merged, aligned to the human reference genome GRCh38, and quantified using the Gencode v.40 human annotation, which specifically included small RNA annotations such as snoRNAs, tRNAs, snRNAs, vault-RNAs, miRNAs, pre-miRNAs, mt-tRNAs, mt-RNAs, piRNAs, and lincRNAs. The piRNAs and tRNAs were retrieved from the piRNAdb^34^ and GtRNAdb 2.0,^133^ respectively.

For a more specialized focus on miRNAs, we used miRDeep2 and downloaded the human and mouse mature miRNA sequences, as well as the hairpin sequences, from the miRbase repository (www.mirbase.org.^134^; the alignment and counting of miRNAs and precursor miRNAs were performed using the default parameters of miRDeep2, with the “t” parameter set to human.

Additionally, a tRNA analysis was carried out using tRAX (https://trna.ucsc.edu/tRAX) with the default parameters, including the construction of reference annotations (tRNA reference DB) allowing the analyses of tRNA fragments and read coverage for each tRNA transcript in four categories—transcript-specific, isodecoder-specific, isotype-specific, and non-specific—that provide precise results on the level of uniqueness of the aligned reads.

Differential gene expression analysis was conducted using the edgeR package.^135^ Following the estimation of common dispersion parameter using the estimateCommonDisp function, differential expression analysis was conducted using pairwise application of the exactTest function for differences between two groups. Genes that were differentially expressed in at least one comparison with a false discovery rate (FDR)-corrected *p* value <0.05 and a log_2_ fold change (FC) >1 were included for the generation of heat maps. Multidimensional scaling was performed on library size normalized count data using the plotMDS function for two-dimensional visualization of similarity between expression profiles.

### miRNA target prediction

*In silico* target prediction of differentially expressed miRNAs was performed using the DIANA-microT online resource.^136^ miRNA::mRNA interactions with an Interaction Score threshold of 0.7 were queried for the individual miRNAs and manually analyzed for overlapping miRNA targets. Genes that were predicted to be targeted by at least 50% of up- or downregulated miRNAs were further analyzed using PPI and pathway analyses.

### PPI and pathway analyses

PPIs were analyzed for differentially expressed protein-coding genes and predicted miRNA targets using the STRING Database version 12.0 (http://www.string-db.org).^137^ Interaction networks were generated using medium confidence scores (minimum required: 0.40) and clustered using Markov Chain Algorithm (MCL) clustering (inflation parameter: 3). Disconnected nodes were hidden from the network.

Functional enrichment and pathway analyses were also performed using the STRING Database version 12.0. Classification systems tested were Gene Ontology (GO) with the sub-ontologies cellular component (GO:CC), biological process (GO:BP) and molecular function (GO:MF), KEGG, Reactome, WikiPathways, and UniProt Keywords functional annotation spaces. Pathway enrichment was analyzed employing Fisher’s exact test followed by FDR correction for multiple testing. Only enriched pathways with FDR corrected *p* values <0.05 are reported.

### Validation of gene candidates by RT-qPCR

RT of total RNA was performed using SuperScript IV VILO kit (Thermo Fisher Scientific) and according to the kit instruction’s manual with a few modifications. Briefly, 500 ng of total RNA extract was reversed transcribed with the following conditions: 10 min incubation at 25°C for annealing; 10 min at 50°C for RT; 5 min at 85°C for enzyme inactivation; and at 4°C for cooling. Samples were diluted with nuclease-free water and subsequently analyzed for qPCR by employing LUNA mastermix (New England Biolabs) with the following parameters: 2 min at 50°C; 1 min at 95°C for initial heat activation; 15 s at 95°C for denaturation; 30 s at 60°C for combined annealing and extension; and 40 cycles.

We also employed miScript II RT kit (QIAGEN) for RT, according to manufacturer’s instruction, specifically for mature miRNA candidates using the HiSpec buffer; whereas, we employed the HiFlex buffer for other small ncRNA candidates. Briefly, 500 ng of total RNA extract was reversed transcribed with the following conditions: 60 min at 37°C for annealing and RT; 5 min at 95°C for enzyme inactivation; and at 4°C for cooling. RT products were analyzed by qPCR as described previously. Ct values > 38 were excluded and data were normalized using the average of two reference genes.

For normalization of data, GAPDH and HPRT1 were employed as reference genes for protein-coding genes. For snoRNAs, SNORD100 and 5.8s rRNA were used; whereas, for other ncRNAs, GAPDH and 5.8s rRNA were employed. The list of primers for validation were indicated in Table S17. The relative fold expression of each candidate gene was expressed as 2^−ΔΔCt^, with ΔΔCt = ΔCt_(infected)_ − ΔCt_(uninfected)_.^138^ To determine differentially expressed genes, normalized Ct or delta Ct (ΔCt_(infected or uninfected)_ = Ct _(target gene)_ − Ct _(average of 2× reference genes)_) values were obtained and the statistical analysis was performed from each group, normalized with the same reference genes, employing GraphPad Prism v.9 using two-way ANOVA with Tukey’s post hoc multiple comparisons test, unless otherwise stated.

### Northern blot analysis

Total RNA extracts from four independent samples were pooled together and an equal amount of RNA (4 μg) from uninfected (control) and SARS-CoV-2-infected (Omicron BA.2 or Delta) NHBE cells was size-fractionated using denaturing polyacrylamide gel electrophoresis (PAGE) as described in a study by Ranches et al.^90^ Samples were heat-denatured for 3 min at 95°C in RNA loading dye (NEB) and size-fractionated on an 8% polyacrylamide gel containing 7 M urea. RNA bands were stained with ethidium bromide, visualized by a Transilluminator (Bio-Rad) and transferred onto a Hybond N^+^ nylon membrane (GE Healthcare). The RNA-membrane crosslinking was carried out at 120 mJ by an ultraviolet (UV) crosslinker (BLX-254, Bio-Link). The oligonucleotide probes complementary to 5′-tRF^Glu(TTC)^, 5′-tRF^Lys(TTT)^, U2 snRNA, 7SL1 RNA, vtRNA 1–2, and RNY4 (Integrated DNA Technologies) (Table S17) were labeled with radioactive gamma [γ-^32^P]-ATP (Hartmann Analytics) using T4 polynucleotide kinase (NEB) and according to manufacturer’s instructions. The membrane was pre-hybridized with the hybridization buffer only, incubated with the probe at 42°C overnight, and washed three times in saline sodium citrate (SSC) (20× SSC: 3 M NaCl and 0.3 M sodium citrate, pH 7.0) buffer with different stringency (2× SSC, twice and 1× SSC, once) for 5 min each with constant shaking at room temperature. The radioactive signal was measured using a Typhoon PhosphorImager (GE Healthcare) and the images were analyzed using ImageJ software. For stripping the probe, the membrane was washed in 0.5% SDS for 1 h and subsequently in distilled water for 30 min at 65°C. The background radioactive signal was checked by PhosphorImager prior to hybridization of the next probe.

## Data and code availability

Raw and processed sequencing data are available in the Gene Expression Omnibus (GEO) repository under accession no. GSE276241. Additional raw data are available from the corresponding authors upon request.

## Acknowledgments

This research was funded in part by the Austrian Science Fund (FWF) 10.55776/P32612 and 10.55776/TAI411 to A.H., 10.55776/P34070 to W.P., and 10.55776/P33510 to D.W. For open access purposes, the authors have applied a CC BY public copyright license to any author accepted manuscript version arising from this submission. We would like to thank Prof. Matthias Erlacher (Institute of Genomics and RNomics, Medical University of Innsbruck) for helpful discussions and critically reading the manuscript, and Asst. Prof. Anne Krogsdam and Martina Hoelzl (MultiOmics core facility, Institute of Bioinformatics, Medical University of Innsbruck) for facilitating the RNA-seq of samples.

## Author contributions

A.H. conceptualized and supervised the study; acquired the funding; and wrote, reviewed, and edited the manuscript. G.R. performed the experiments and analysis of data and wrote, reviewed, and edited the manuscript. H.H. performed sequencing data analysis and wrote, reviewed, and edited the manuscript. V.Z. and M.P. performed the experiments. W.P. and D.W. acquired the funding and wrote, reviewed, and edited the manuscript. K.K performed sequencing data analysis and wrote, reviewed, and edited the manuscript.

## Declaration of interests

The authors declare no competing interests.

## Supplemental information

Data S1. Tables S6, S8, S12, S15, and S17

Document S2. Article plus supplemental information
