# Supplementary material for: Differentially expressed ncRNAs as key regulators in infection of human bronchial epithelial cells by the SARS-CoV-2 Delta variant
Source: Mol Ther Nucleic Acids. 2025 May 14;36(2):102559. doi: 10.1016/j.omtn.2025.102559 (PMC12162024; doi:10.1016/j.omtn.2025.102559)
Supplement: Document S1. Figures S1–S4, and Tables S1–S5, S7, S9–S11, S13, S14, and S16 [file mmc1.pdf]

OMTN, Volume *36*

Supplemental information

Differentially expressed ncRNAs as key regulators in infection of human bronchial epithelial cells by the SARS-CoV-2 Delta variant

Glory Ranches, Hubert Hackl, Viktoria Zaderer, Melanie Ploner, Wilfried Posch, Doris Wilflingseder, Kai Kummer, and Alexander Hüttenhofer

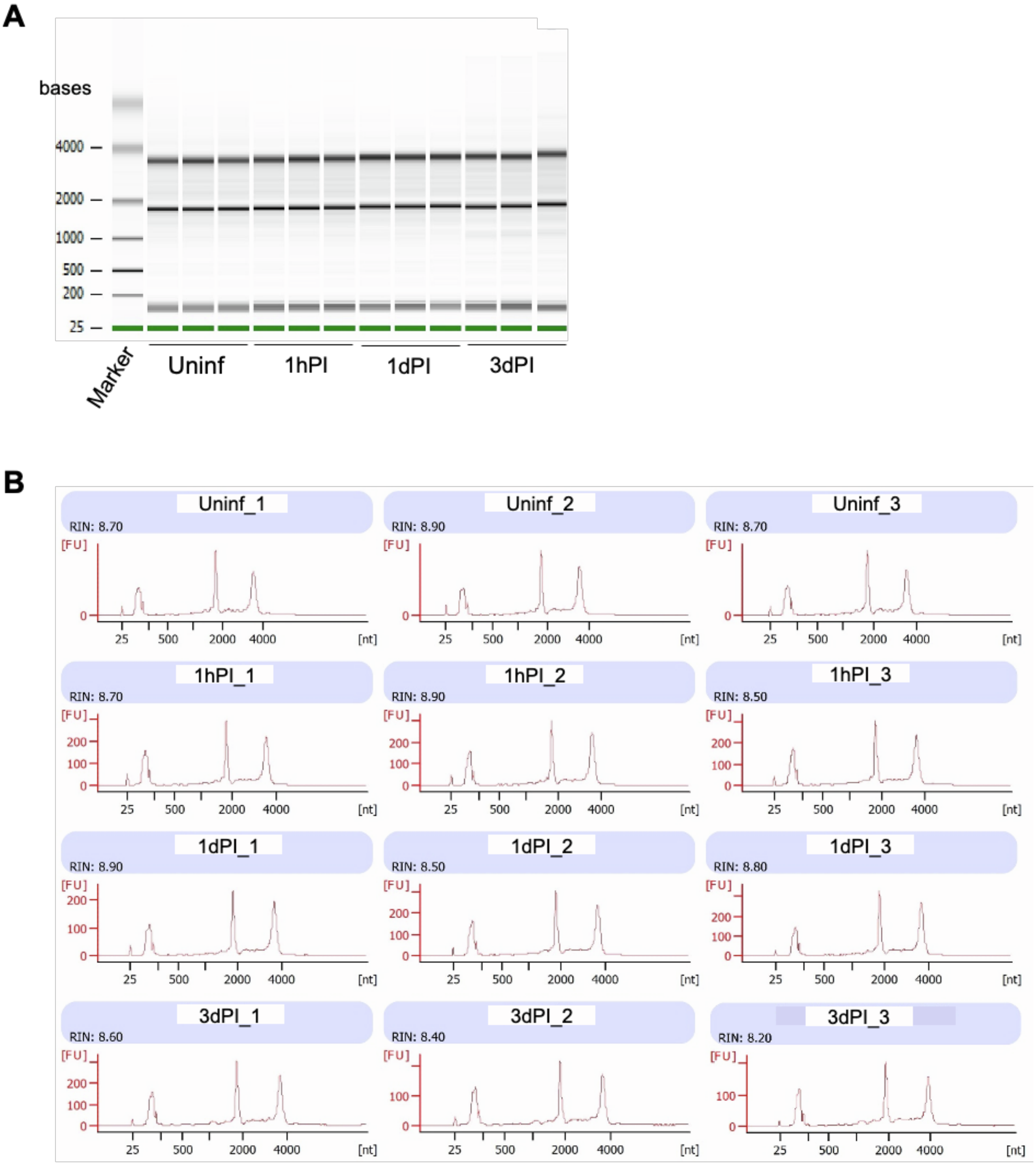

Figure S1. Bioanalyzer analysis of total RNA extracts from SARS-CoV-2-infected and uninfected NHBE cells using RNA pico chip. A) PAGE gel profile of total RNA extracts from either uninfected (Uninf) or SARS-CoV-2 infected cells for 1 hr (1hPI), 24 hrs (1dPI) and 72 hrs (3dPI) showing the distribution and molecular size of RNA populations for each sample. B) Electropherogram profile of total RNA extracts in (A). The experiment was performed using three independent samples.

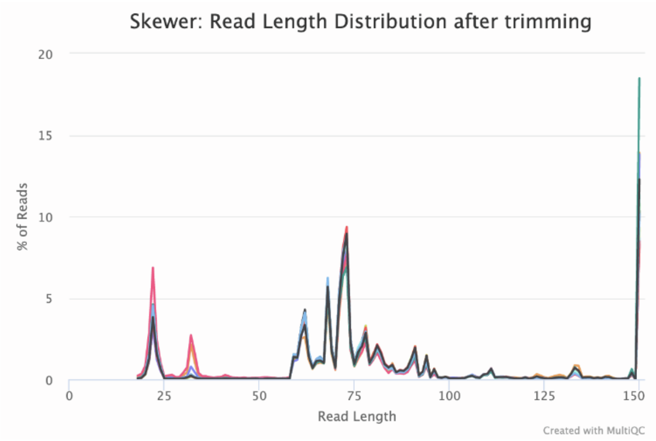

Figure S2. Analysis of the length distribution of RNA-seq reads following adapter trimming by Skewer [1]. The abundance (% Reads) of RNA populations or fragments in uninfected and SARS-CoV-2 infected NHBE cells is shown.

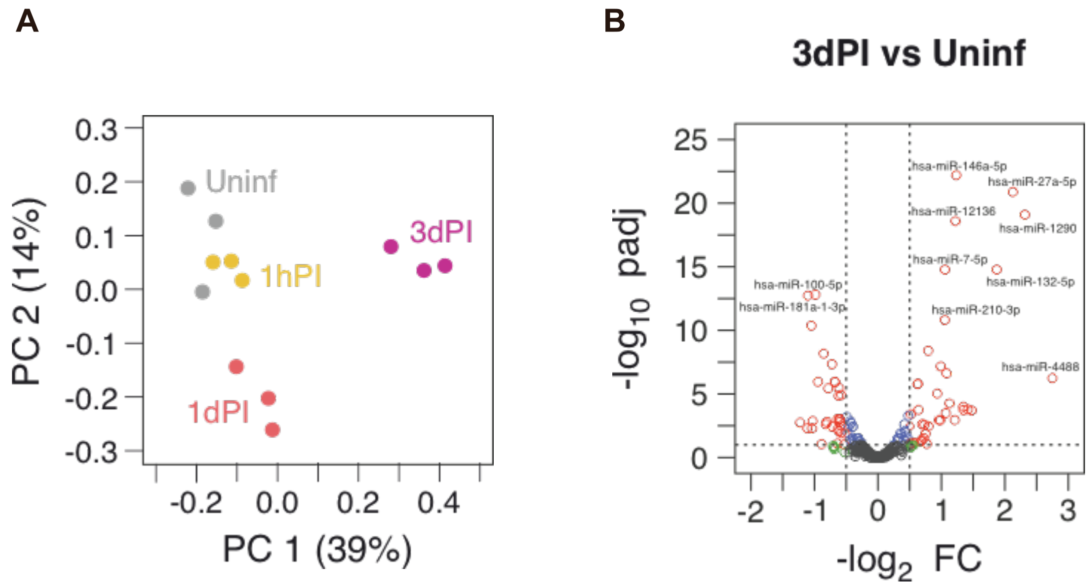

Figure S3. Sequencing and differential gene expression analysis of microRNAs. A) Principal component analysis of differentially expressed miRNAs in NHBE cells upon SARS-CoV-2 infection following 1-hr post-infection (1hPI), 1-day (24 hr) post-infection (1dPI) and 3-day (72 hr) post-infection (3dPI). Uninfected (Uninf) NHBE cells were used as control group. B) Volcano plot analysis of –log2 fold change (FC) versus –log10 padj between 3dPI and Uninf.

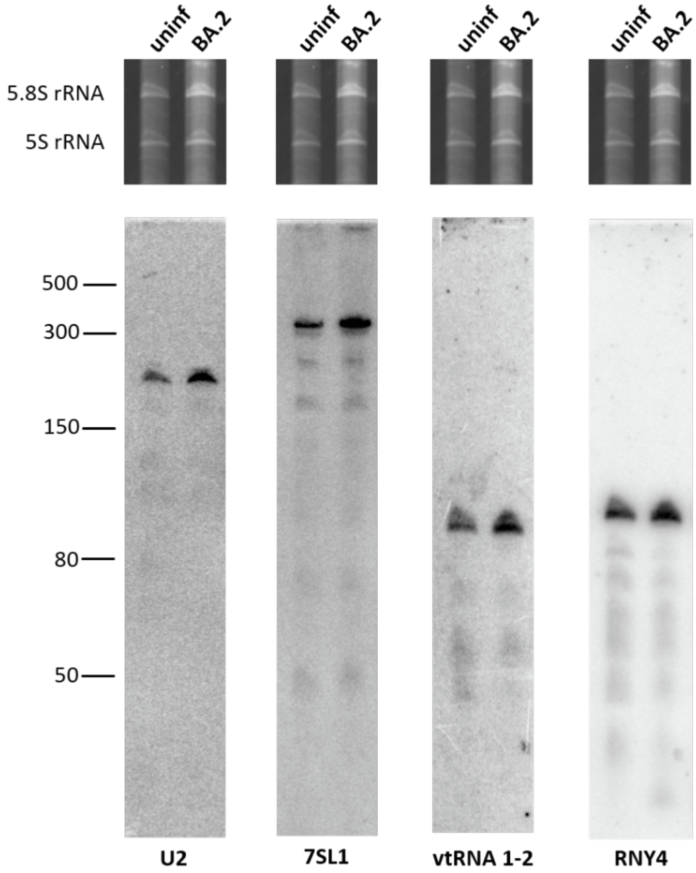

Figure S4. Northern blot analysis of U2, 7SL1, vtRNA 1-2 and RNY4 ncRNAs in uninfected (uninf) versus Omicron BA.2 (BA.2) infected NHBE cells. Increased abundance of all ncRNAs in Omicron BA.2 infected cells is shown; upon longer exposure of northern blots, also smaller ncRNA fragments at low abundance are evident which, however, do not differ in their abundance in BA.2 infected versus uninfected cells, in contrast to full length RNAs.

Table S1. Differentially expressed protein-coding genes in SARS-CoV-2 infected (3dPI) vs. uninfected NHBE cells.

Gene	logFC	logCPM	p-value (FDR)
CXCL10	10.18	0.94	1.23E-28
NCOA7	7.58	2.78	1.39E-39
IFIT2	7.50	3.66	6.94E-44
IFIT1	7.28	1.07	3.16E-27
SIX3	6.86	2.89	4.16E-39
NT5C3A	5.81	0.92	5.31E-22
ZC3HAV1	4.58	3.12	1.22E-24
EIF2AK2	4.54	1.12	2.03E-18
TRIM25	4.39	1.34	1.07E-19
BIRC3	4.17	1.69	2.34E-19
PAQR5	3.37	3.33	2.05E-16
DDX3X	2.51	1.63	4.40E-09
RPL27A	2.43	1.72	8.94E-09
MTRNR2L8	2.32	6.24	1.65E-09
GAN	2.22	1.53	1.97E-07
SLFN5	2.18	1.24	7.60E-07
TXNIP	2.08	1.88	8.21E-07
ARRDC3	1.93	1.81	5.69E-06
MTRNR2L12	1.35	3.94	9.52E-04
EPM2AIP1	-1.70	4.54	1.72E-05
KLF5	-2.19	5.42	1.80E-08
CD24	-2.55	2.34	2.44E-09

Table S2. Pathway analysis of differentially expressed mRNAs.

#category	term ID	term description	observed gene count	background gene count (whole genome)	strength	p-value (FDR corrected)
GO Biological Process	GO:0009615	Response to virus	9	356	1.27	1.26E-05
GO Biological Process	GO:0051607	Defense response to virus	7	252	1.31	0.00035
GO Biological Process	GO:0009607	Response to biotic stimulus	11	1375	0.77	0.0035
GO Biological Process	GO:1903900	Regulation of viral life cycle	5	139	1.42	0.0043
GO Biological Process	GO:0044419	Biological process involved in interspecies interaction between organisms	11	1490	0.73	0.0052
GO Biological Process	GO:0045069	Regulation of viral genome replication	4	86	1.53	0.0113
GO Biological Process	GO:0051707	Response to other organism	10	1328	0.74	0.0113
GO Biological Process	GO:0048525	Negative regulation of viral process	4	94	1.49	0.0131
GO Biological Process	GO:0065009	Regulation of molecular function	14	3085	0.52	0.0185
GO Biological Process	GO:0032728	Positive regulation of interferon-beta production	3	41	1.73	0.0352
GO Biological Process	GO:0010941	Regulation of cell death	10	1651	0.65	0.0433
GO Biological Process	GO:0098542	Defense response to other organism	8	989	0.77	0.0433
KEGG	hsa04622	RIG-I-like receptor signaling pathway	3	69	1.5	0.043
Reactome	HSA-1169408	ISG15 antiviral mechanism	4	74	1.6	0.0078
Reactome	HSA-1280215	Cytokine Signaling in Immune system	8	706	0.92	0.0078
Reactome	HSA-168256	Immune System	12	1979	0.65	0.0078
Reactome	HSA-913531	Interferon Signaling	5	199	1.26	0.0078
Reactome	HSA-168255	Influenza Infection	4	155	1.27	0.0235
WikiPathways	WP619	Type II interferon signaling	3	37	1.77	0.0171
WikiPathways	WP3865	Novel intracellular components of RIG-I-like receptor pathway	3	59	1.57	0.032
WikiPathways	WP5039	SARS-CoV-2 innate immunity evasion and cell-specific immune response	3	66	1.52	0.032
UniProt Keywords	KW-0051	Antiviral defense	5	127	1.46	0.0006
UniProt Keywords	KW-0399	Innate immunity	6	329	1.12	0.0017
UniProt Keywords	KW-0832	Ubl conjugation	12	2399	0.56	0.0071
UniProt Keywords	KW-0945	Host-virus interaction	6	540	0.91	0.0129

Table S3. Pathway analysis of differentially expressed mRNA subclusters.

#category	term ID	term description	observed gene count	background gene count (whole genome)	strength	p-value (FDR corrected)
GO Biological Process	GO:0051607	Defense response to virus	5	252	1.89	5.70E-06
GO Biological Process	GO:0045087	Innate immune response	5	754	1.42	0.00033
GO Biological Process	GO:0045071	Negative regulation of viral genome replication	3	56	2.32	0.0008
GO Biological Process	GO:0034097	Response to cytokine	4	804	1.29	0.0125
GO Biological Process	GO:0035455	Response to interferon-alpha	2	22	2.55	0.0125
GO Biological Process	GO:0140374	Antiviral innate immune response	2	22	2.55	0.0125
KEGG	hsa05160	Hepatitis C	3	157	1.88	0.0017
Reactome	HSA-1280215	Cytokine Signaling in Immune system	4	706	1.35	0.0185
Reactome	HSA-913531	Interferon Signaling	3	199	1.77	0.0185
WikiPathways	WP619	Type II interferon signaling	3	37	2.5	6.04E-05
WikiPathways	WP5039	SARS-CoV-2 innate immunity evasion and cell-specific immune response	2	66	2.08	0.0455
UniProt Keywords	KW-0051	Antiviral defense	4	127	2.09	6.24E-06
UniProt Keywords	KW-0399	Innate immunity	4	329	1.68	0.00013
UniProt Keywords	KW-0694	RNA-binding	4	686	1.36	0.0012
KEGG	hsa03010	Ribosome	2	131	2.18	0.0152
Reactome	HSA-156827	L13a-mediated translational silencing of Ceruloplasmin expression	2	110	2.25	0.0471
Reactome	HSA-156902	Peptide chain elongation	2	88	2.35	0.0471
Reactome	HSA-1799339	SRP-dependent cotranslational protein targeting to membrane	2	110	2.25	0.0471
Reactome	HSA-192823	Viral mRNA Translation	2	88	2.35	0.0471
Reactome	HSA-2408557	Selenocysteine synthesis	2	92	2.33	0.0471
Reactome	HSA-6791226	Major pathway of rRNA processing in the nucleolus and cytosol	2	181	2.04	0.0471
Reactome	HSA-72689	Formation of a pool of free 40S subunits	2	100	2.29	0.0471
Reactome	HSA-72706	GTP hydrolysis and joining of the 60S ribosomal subunit	2	111	2.25	0.0471
Reactome	HSA-72764	Eukaryotic Translation Termination	2	92	2.33	0.0471
Reactome	HSA-9010553	Regulation of expression of SLITs and ROBOs	2	169	2.07	0.0471
Reactome	HSA-9633012	Response of EIF2AK4 (GCN2) to amino acid deficiency	2	100	2.29	0.0471
Reactome	HSA-975956	Nonsense Mediated Decay (NMD) independent of the Exon Junction Complex (EJC)	2	94	2.32	0.0471
Reactome	HSA-975957	Nonsense Mediated Decay (NMD) enhanced by the Exon Junction Complex (EJC)	2	114	2.24	0.0471
WikiPathways	WP477	Cytoplasmic ribosomal proteins	2	88	2.35	0.0161
GO Biological Process	GO:1900118	Negative regulation of execution phase of apoptosis	2	20	2.99	0.0187
GO Molecular Function	GO:0048019	Receptor antagonist activity	2	30	2.82	0.0126

Table S4. Differentially expressed miRNAs in SARS-CoV-2-infected NHBE cells (3dPI vs. Uninfected).

Gene	logFC	logCPM	p-value (FDR)
hsa-miR-155-5p	3.57	5.42	2.83E-87
hsa-miR-4488	2.75	2.11	5.63E-07
hsa-miR-1290	2.31	4.17	8.10E-20
hsa-miR-27a-5p	2.13	3.91	1.29E-21
hsa-miR-132-5p	1.87	3.81	1.68E-15
hsa-miR-1246	1.85	6.66	2.81E-37
hsa-miR-17-3p	1.48	2.59	2.01E-04
hsa-miR-143-3p	1.42	2.32	1.64E-04
hsa-miR-1303	1.35	2.62	1.87E-04
hsa-miR-147b-3p	1.34	2.27	1.03E-04
hsa-miR-146a-5p	1.24	12.10	6.22E-23
hsa-miR-12136	1.22	7.43	2.58E-19
hsa-miR-7704	1.21	2.11	1.14E-03
hsa-miR-324-3p	1.13	3.09	5.71E-05
hsa-miR-33b-5p	1.08	4.35	2.39E-07
hsa-miR-212-3p	1.07	3.25	3.28E-04
hsa-miR-7-5p	1.06	8.12	1.68E-15
hsa-miR-210-3p	1.05	5.84	1.55E-11
hsa-miR-576-3p	-1.03	3.18	1.36E-03
hsa-miR-874-3p	-1.04	2.91	4.95E-03
hsa-miR-181a-2-3p	-1.05	5.86	4.30E-11
hsa-miR-181a-1-3p	-1.10	6.16	1.90E-13
hsa-miR-100-3p	-1.11	2.73	5.05E-03
hsa-miR-874-5p	-1.23	2.08	1.80E-03

Table S5. Pathway enrichment of upregulated microRNA targets.

#category	term ID	term description	observed gene count	background gene count (whole genome)	strength	p-value (FDR corrected)
GO Biological Process	GO:0051171	Regulation of nitrogen compound metabolic process	61	5734	0.25	0.0038
GO Biological Process	GO:0051252	Regulation of RNA metabolic process	45	3759	0.3	0.0098
GO Biological Process	GO:0080090	Regulation of primary metabolic process	60	5899	0.23	0.0098
GO Biological Process	GO:0019219	Regulation of nucleobase-containing compound metabolic process	46	4074	0.28	0.0181
GO Biological Process	GO:0031323	Regulation of cellular metabolic process	57	5681	0.22	0.0181
GO Biological Process	GO:0060255	Regulation of macromolecule metabolic process	61	6249	0.21	0.0181
GO Biological Process	GO:0007399	Nervous system development	30	2188	0.36	0.0246
GO Biological Process	GO:0010468	Regulation of gene expression	51	4899	0.24	0.0246
GO Biological Process	GO:0019222	Regulation of metabolic process	63	6784	0.19	0.0345
GO Biological Process	GO:0007275	Multicellular organism development	45	4209	0.25	0.0417
GO Cellular Component	GO:0030424	Axon	17	651	0.64	0.00092
GO Cellular Component	GO:0005654	Nucleoplasm	48	4169	0.28	0.0014
GO Cellular Component	GO:0005634	Nucleus	70	7672	0.18	0.0042
GO Cellular Component	GO:0031981	Nuclear lumen	49	4526	0.26	0.0042
GO Cellular Component	GO:0043005	Neuron projection	23	1391	0.44	0.0042
GO Cellular Component	GO:0036477	Somatodendritic compartment	17	855	0.52	0.0054
GO Cellular Component	GO:0070013	Intracellular organelle lumen	55	5660	0.21	0.0091
GO Cellular Component	GO:0032839	Dendrite cytoplasm	4	30	1.35	0.0099
GO Cellular Component	GO:0000785	Chromatin	20	1285	0.41	0.0156
GO Cellular Component	GO:0030425	Dendrite	13	624	0.54	0.0178
GO Cellular Component	GO:0043197	Dendritic spine	7	176	0.82	0.0178
GO Cellular Component	GO:0098794	Postsynapse	13	621	0.54	0.0178
GO Cellular Component	GO:0030054	Cell junction	26	2115	0.31	0.0362
UniProt Keywords	KW-0597	Phosphoprotein	79	8122	0.21	1.14E-05
UniProt Keywords	KW-0025	Alternative splicing	88	10313	0.15	0.00022
UniProt Keywords	KW-0539	Nucleus	56	5278	0.25	0.0003
UniProt Keywords	KW-0862	Zinc	33	2347	0.37	0.0003
UniProt Keywords	KW-0863	Zinc-finger	28	1749	0.43	0.0003
UniProt Keywords	KW-0479	Metal-binding	41	3702	0.27	0.0037
UniProt Keywords	KW-0832	Ubl conjugation	30	2399	0.32	0.0064
UniProt Keywords	KW-0678	Repressor	12	611	0.52	0.0267
UniProt Keywords	KW-0804	Transcription	28	2369	0.3	0.0267
UniProt Keywords	KW-0160	Chromosomal rearrangement	8	312	0.63	0.0447

Table S6. Pathway enrichment for subclusters of upregulated microRNA targets.

see Supplemental Spreadsheets

Table S7. Pathway enrichment of downregulated microRNA targets.

#category	term ID	term description	observed gene count	background gene count (whole genome)	strength	p-value (FDR corrected)
UniProt Keywords	KW-0025	Alternative splicing	35	10313	0.19	0.05

Table S8. Pathway enrichment for subclusters of downregulated microRNA targets.

see Supplemental Spreadsheets

Table S9. Differentially expressed piRNAs in SARS-CoV-2-infected (3dPI) vs uninfected NHBE cells.

Gene	logFC	logCPM	p-value (FDR)
hsa-piR-33005	8.08	5.61	1.16E-28
hsa-piR-23019	5.20	6.38	6.63E-16
hsa-piR-23020	4.93	5.01	1.50E-14
hsa-piR-1079	4.12	4.72	5.66E-11
hsa-piR-20923	3.36	2.31	1.39E-07
hsa-piR-32186	3.12	3.30	5.38E-07
hsa-piR-28060	3.12	2.07	1.10E-06
hsa-piR-12655	3.10	11.78	4.14E-07
hsa-piR-6960	3.09	6.91	4.43E-07
hsa-piR-23137	3.05	4.86	6.74E-07
hsa-piR-27306	3.01	2.60	1.84E-06
hsa-piR-32899	2.95	2.15	2.39E-06
hsa-piR-32157	2.85	10.66	2.73E-06
hsa-piR-32952	2.80	3.59	5.65E-06
hsa-piR-32995	2.74	9.14	6.70E-06
hsa-piR-27429	2.60	2.87	2.90E-05
hsa-piR-1044	2.49	12.22	4.64E-05
hsa-piR-24541	2.31	11.57	1.78E-04
hsa-piR-11621	2.13	5.51	6.83E-04
hsa-piR-32946	1.97	3.28	2.36E-03
hsa-piR-1028	1.93	6.47	2.62E-03
hsa-piR-28521	1.84	3.68	4.77E-03
hsa-piR-7005	1.72	1.52	0.013
hsa-piR-33041	1.68	9.25	0.010
hsa-piR-23997	1.47	4.80	0.033
hsa-piR-23140	1.46	4.11	0.035
hsa-piR-23495	1.46	3.85	0.035
hsa-piR-32882	1.43	2.25	0.046
hsa-piR-33070	-1.69	3.35	0.012
hsa-piR-32835	-1.80	5.14	5.42E-03

Table S10. Predicted targets of differentially expressed piRNAs.

piRNA	regulation	predicted targets [1]
hsa-piR-33005	up	TFCP2L1
hsa-piR-23019	up	-
hsa-piR-23020	up	-
hsa-piR-1079	up	-
hsa-piR-20923	up	LINC02558
hsa-piR-32186	up	DPF2
hsa-piR-28060	up	SHF
hsa-piR-12655	up	-
hsa-piR-6960	up	-
hsa-piR-23137	up	-
hsa-piR-27306	up	LINC00317
hsa-piR-32899	up	-
hsa-piR-32157	up	IFT88, CRTC1, FAM83C, HAUS8, LINC01132, OTUB2, PPM1K-DT
hsa-piR-32952	up	-
hsa-piR-32995	up	-
hsa-piR-27429	up	BIRC5
hsa-piR-1044	up	LINC00317
hsa-piR-24541	up	-
hsa-piR-11621	up	LOC101928075, SNAP25-AS1
hsa-piR-32946	up	-
hsa-piR-1028	up	-
hsa-piR-28521	up	-
hsa-piR-7005	up	-
hsa-piR-33041	up	GVQW3, HTT, LINC02085
hsa-piR-23997	up	-
hsa-piR-23140	up	-
hsa-piR-23495	up	-
hsa-piR-32882	up	-
hsa-piR-23511	up	-
hsa-piR-32897	up	CYP2W1, SIAE
hsa-piR-33182	up	-
hsa-piR-33070	down	TMEM53
hsa-piR-32835	down	C20orf194

[1] piRNAdb.org

Table S11. Differentially expressed nuclear (top) and mitochondrial (bottom) tRNAs in SARS-CoV-2-infected NHBE cells (3dPI vs. Uninfected).

Gene	logFC	logCPM	p-value (FDR)
His-tRNA	3.50	5.97	1.46E-18
Gly-tRNA	2.70	12.60	2.56E-12
Val-tRNA	2.46	7.74	1.59E-10
SeC-tRNA	2.33	2.69	5.07E-09
Glu-tRNA	2.16	11.62	1.73E-08
Lys-tRNA	1.91	6.94	7.25E-07
Asp-tRNA	1.69	7.78	1.42E-05
Pro-tRNA	1.58	3.15	8.88E-05
Met-tRNA	1.58	2.40	1.67E-04
Tyr-tRNA	1.41	5.09	4.48E-04
Cys-tRNA	1.34	2.25	1.97E-03
MT-TM	2.86	8.52	1.50E-13
MT-TC	2.85	2.30	7.35E-12
MT-TS1	2.40	6.22	4.51E-10
MT-TY	-1.76	4.33	9.29E-06

Table S12. Differentially expressed tRNA fragments in SARS-CoV-2-infected NHBE cells (3dPI vs. Uninfected).

see Supplemental Spreadsheets

Table S13. Differentially expressed snoRNAs in SARS-CoV-2-infected (3dPI) vs uninfected NHBE cells.

Gene	logFC	logCPM	p-value (FDR)
SNORD3A	2.30	6.77	0.00019
SNORD118	2.10	3.71	0.0010
SNORA81	1.21	5.84	0.0034
SNORA62	1.05	3.64	0.018
SNORA63.7	-1.01	2.02	0.044
SNORD116-11	-1.10	2.25	0.022
SNORD11B	-1.11	5.42	0.0093
SNORD116-13	-1.21	5.75	0.0036
SCARNA1	-1.49	7.87	0.00016
SNORD116-5	-1.58	9.79	0.017
SNORD113.6	-1.96	1.76	0.0043
SNORD115-12	-10.56	3.85	9.14E-21
SNORD115-18	-11.38	5.12	2.29E-35
SNORD116-3	-15.29	8.10	1.59E-43

Table S14. Differentially expressed Y RNAs, vtRNAs and lincRNAs in SARS-CoV-2-infected (3dPI) vs uninfected NHBE cells.

Gene	logFC	logCPM	p-value (FDR)
RNY3P2	4.27	2.07	6.46E-21
RNY3P8	4.26	2.07	1.02E-20
RNY4P25	3.98	2.77	3.19E-20
RNY4P18	3.96	2.75	4.18E-20
RNY4P20	3.74	6.02	1.78E-20
RNY4P7	3.35	6.43	2.50E-17
RP11-403P17.4	2.89	2.72	1.42E-12
RNY4P10	2.43	7.16	2.57E-10
NEAT1	2.04	2.98	3.50E-07
RNY4	1.84	10.02	1.83E-06
VTRNA1-2	1.82	2.69	6.70E-06
NORAD	1.77	2.00	2.47E-05
VTRNA2-1	1.70	1.46	7.51E-05
RNY1	1.50	10.29	0.00013
VTRNA1-1	1.34	5.06	0.00090
MALAT1	1.00	4.61	0.025

Table S15. Differentially expressed Y RNA transcripts in SARS-CoV-2-infected (3dPI) vs uninfected NHBE cells.

see Supplemental Spreadsheets

Table S16. Host target genes of differentially expressed snoRNAs in SARS-CoV-2-infected NHBE cells.

SnoRNA	RNA target	Host gene[1]	Correlation[2]
SNORA6	28S rRNA	RPSA (LAMR1): Viral receptor	0.17
SNORA46	18S rRNA	CNOT1: mRNA turnover,	0.56
SNORA46		AHSA1:interacts with HSP90	-0.10
SNORA51	Orphan snoRNA	NOL5A (NOP56), N4BP2, WDR17, KLHL23	0.36
SNORA62	28S rRNA	RPSA (LAMR1): viral receptor	0.25
SNORA63	28S rRNA	HSPA9 (mitochondrial heat shock protein 70)	-0.07
SNORA74A	28S rRNA, U3 snRNA	MATR3 (innate immune response)	-0.29
SNORA80A	Orphan snoRNA	URB1	-0.17
SNORA81	Orphan snoRNA	EIF4A2	0.03
SNORD3A (U3)	18S rRNA	none	--
SNORD11B	18S rRNA	NOP58	-0.27
SNORD20	18S rRNA	NCL (nucleolin)	-0.33
SNORD97	$tRNA^{Met}$	EIF4G2 (translation at non-AUG codons)	0.80**
SNORD109A	Orphan snoRNA	SNRPN-PWS transcript (maternally imprinted)	0.12
SNORD109B	Orphan snoRNA	SNRPN-PWS transcript (maternally imprinted)	0.12
SNORD113.6	Orphan snoRNA	MEG8 (maternally imprinted)	--
SNORD115	Orphan snoRNA	SNRPN-PWS transcript (maternally imprinted)	0.39
SNORD116	Orphan snoRNA	SNRPN-PWS transcript (maternally imprinted)	0.36
SNORD118	28S rRNA	STAB2 (transmembrane protein)	--
SCARNA1	U2 snRNA	PPP1R8 (mRNA splicing)	0.35
SCARNA3	U6 snRNA	RFWD2 (induces cell proliferation)	0.40
SCARNA12	U5 snRNA	PHB2 (involved in RIG-1 mediated signal transduction)	0.06

[1] Adapted from snOPY database:
http://snoopy.med.miyazaki-u.ac.jp/

[2] Spearman rank based correlation coefficient (ρ) between snoRNA and its host gene (protein coding transcript) across all samples based on normalized counts (** p-value<0.01; ρ>0.2 are indicated in gray)

red indicate significant differential expression of both snoRNA and its host gene.

Table S17. List of primers used for validation of candidate genes by qPCR and northern blot.

see Supplemental Spreadsheets

References

1. Jiang, H, Lei, R, Ding, SW, and Zhu, S (2014). Skewer: a fast and accurate adapter trimmer for next-generation sequencing paired-end reads. *BMC Bioinformatics* **15**: 182.
